# ROS Activated NETosis of Bone Marrow CD55^+^ Intermediate Mature Neutrophils Through HIF1α‐PADI4 Pathway to Initiate Bone Aging

**DOI:** 10.1002/advs.202500046

**Published:** 2026-01-08

**Authors:** Yutong Guo, Shengjie Cui, Xi Wen, Yidi Wang, Ben Wu, Yixiang Wang, Yan Gu

**Affiliations:** ^1^ Department of Orthodontics Peking University School and Hospital of Stomatology & National Center for Stomatology & National Clinical Research Center for Oral Diseases & National Engineering Research Center of Oral Biomaterials and Digital Medical Devices& Beijing Key Laboratory of Digital Stomatology & NHC Key Laboratory of Digital Stomatology & NMPA Key Laboratory For Dental Materials Beijing P. R. China; ^2^ Department of General Dentistry Peking University School and Hospital of Stomatology Beijing P. R. China; ^3^ Center For Applied Statistics School of Statistics Renmin University of China Beijing P. R. China; ^4^ Central Laboratory Peking University School and Hospital of Stomatology Beijing P. R. China

**Keywords:** BMSCs senescence, bone aging, CD55^+^ neutrophils, intermediate mature neutrophils, NETosis, ROS

## Abstract

Neutrophil NETosis is markedly dysregulated in the aging body. Bone marrow serves as the powerhouse of neutrophil differentiation, while the state of neutrophil NETosis therein and its relationship with bone aging remains largely elusive. Moreover, it remains unclear how neutrophil heterogeneity and pro‐inflammatory cues within bone marrow synergistically regulate neutrophil NETosis. Here, we find neutrophil NETosis is highly activated in the bone marrow of 3‐mon male senescence‐accelerated mouse prone 6 (SAMP6), and the released NETs induce BMSCs senescence and impairs their osteogenesis. Further, we verify in vivo NETs‐clearance significantly ameliorates bone aging of 3‐mon male SAMP6 mice. Next, through scRNA‐seq we find a CD55^+^ intermediate mature neutrophil subset enriching in the 3‐mon male SAMP6 bone marrow, characterized by significantly upregulated NETosis. Through cell transfer, we demonstrate this subset directly induces bone aging. Mechanistically, elevated ROS within the bone marrow of SAMP6 integrates with a CD55‐primed HIF1ɑ‐PADI4 pathway to trigger NETosis, and senescent BMSCs serve as a ROS‐producer. In summary, our results demonstrate that activated NETosis in CD55^+^ intermediate‐mature neutrophils plays a key role in initiating bone aging. Also, we uncover the vicious cycle of inflammaging between immune dysregulation and cellular senescence in bone marrow, providing potential targets for osteoporosis treatment.

## Graphical abstract

In this study, we find CD55^+^ neutrophils show activated NETosis within bone marrow, induce BMSC senescence and osteogenesis inhibition, finally leading to bone aging initiation. Mechanistically, ROS synergizes with the CD55‐driven HIF1α‐PADI4 pathway to promote NETosis. We also verify senescent BMSCs as a ROS source, forming a vicious cycle of inflammaging with neutrophils within bone marrow.

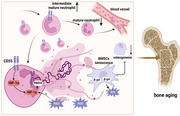

## 1 Introduction

Aging has been recognized as an important driving factor of osteoporosis [1]. In age‐related diseases, cellular senescence and chronic inflammation are two important hallmarks, both of which have been observed in osteoporotic bone marrow (BM) [2, 3]. On one hand, BM mesenchymal stem cells (BMSCs) and osteoblasts undergo senescence, contributing to impaired bone formation [4]. On the other hand, the chronic and low‐grade inflammation termed “inflammaging” develops in BM, resulting from dysregulated immune cells including macrophages, T cells and B cells, as well as senescent cells through SASP (senescence‐associated secretory phenotype) [5].

Neutrophils are the most abundant immune cells within bone marrow, and mounting evidence has demonstrated that dysregulated neutrophil NETosis in aging bodies contributes to various age‐related diseases [6, 7, 8, 9]. Neutrophil extracellular traps (NETs) release, also known as NETosis, is a critical antimicrobial function of neutrophils that helps combat infection and restrict pathogen spread [10]. NETs are unique web‐like structures released to extracellular space by neutrophils, containing decondensed chromatin, citrullinated histone, granular proteins including myeloperoxidase (MPO) and neutrophil elastase (NE) [11]. Recently, NETosis has been found to be activated under sterile and pro‐inflammatory stimuli including ROS and IL‐6, and over‐activated neutrophils can release excessive NETs to damage tissue cells and activate surrounding immune cells [12]. However, the impact of aging on neutrophil NETosis in BM as well as the consequent effects of NETs on the BM microenvironment remains poorly understood.

BM is the site of neutrophil differentiation and harbors a heterogeneous neutrophil population of both immature and mature subsets [6, 13]. Under physiological conditions, immature neutrophils can be less efficient in releasing NETs than mature neutrophils to combat pathogens [14]. However, under pro‐inflammatory conditions, especially systemic inflammation, immature neutrophils are reported to migrate from BM to peripheral tissues and undergo significant NETosis along with other activated immune response [1, 15, 16, 17]. Therefore, within the highly inflammatory BM micro‐environment of aging bodies, whether BM neutrophils can undergo NETosis before entering the circulation, and which neutrophil subset could be the most NETosis‐prone, remains to be elucidated.

In this study, we constructed a femur BM neutrophil atlas and analyzed the dynamics of neutrophil NETosis dynamics in SAMP6 and C57BL6/J mice. We demonstrated that the activated BM neutrophil NETosis instigated bone aging through inducing BMSCs senescence and impairing their osteogenesis. Also, through scRNA‐seq we identified an intermediate mature BM neutrophil subset CD55^+^ neutrophils with abnormally up‐regulated NETosis in aging BM. Adoptive transfer of this subset alone was sufficient to trigger bone aging. Mechanistically, we found elevated ROS in the BM of SAMP6 integrated with CD55‐primed HIF1ɑ‐PADI4 pathway to activate NETosis. Moreover, we identified senescent BMSCs as a source of ROS in the BM, forming a vicious cycle of inflammaging through their interaction with BM neutrophils. This study provides new insights into how environmental cues and cellular heterogeneity synergistically regulate neutrophil NETosis during aging, and also identifies new targets for bone aging treatment.

## 2 Results

### 2.1 Femur Bone Loss and BMSCs Senescence Initiate at 3‐mon in SAMP6

To establish a bone aging model, we used senescence‐accelerated prone 6 (SAMP6) mice, with senescence‐accelerated resistant 1 (SAMR1) mice serving as naturally aging controls.

**FIGURE 1:**
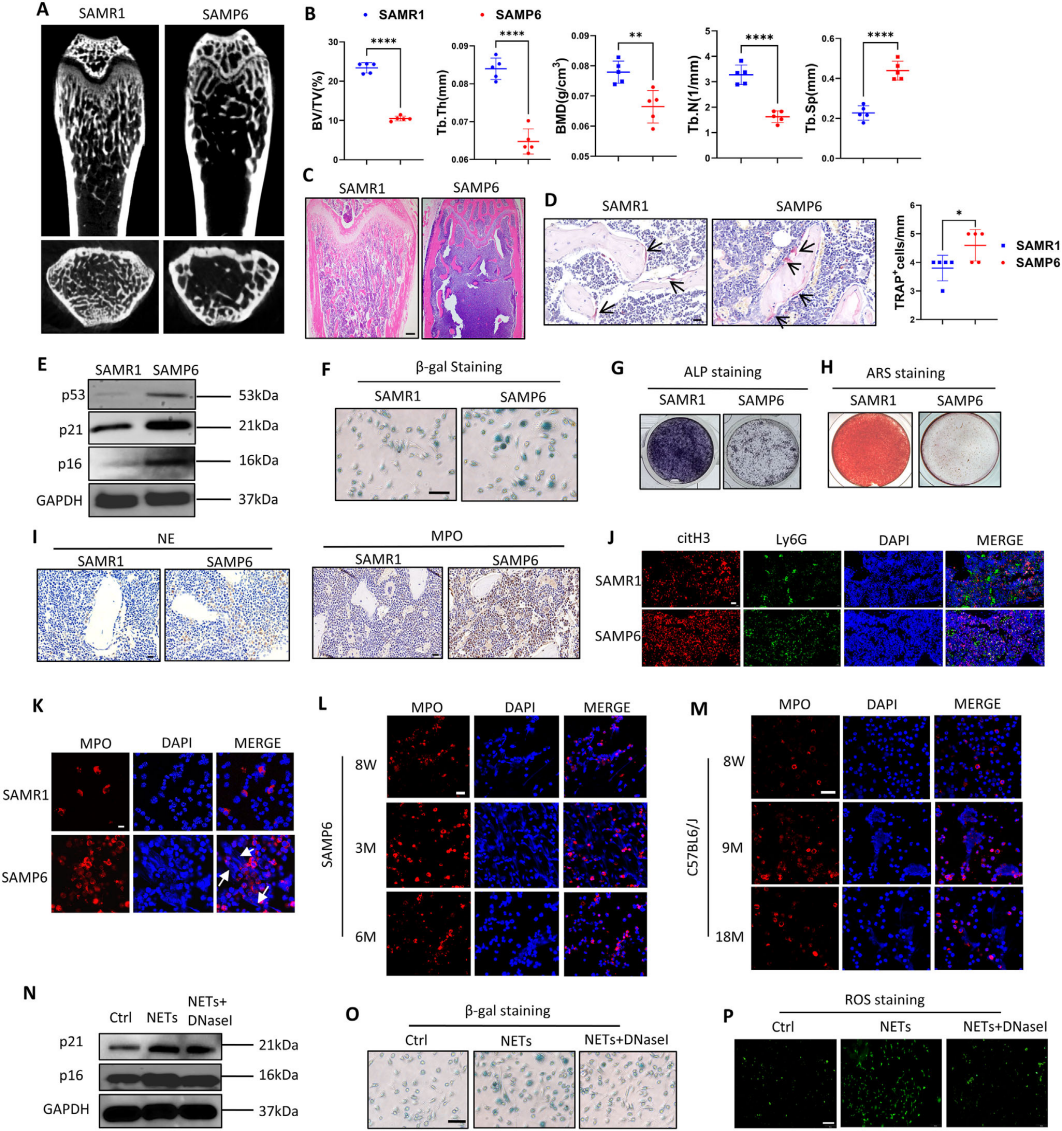
3‐mon male SAMP6 showed femur bone loss and BMSCs senescence compared with SAMR1. (A) µCT images of distal femurs, trabecular bone of 3‐mon male SAMP6 and SAMR1 indicated lower bone mass of SAMP6. (B) Quantitative µCT analysis of distal end of femurs indicated 3‐mon SAMP6 showed significantly lower BMD, BV/TV, Tb.th, Tb.N and increased Tb.Sp than SAMR1. n = 5 mice per group. (C) H&E analysis from the femur metaphysic indicated fewer trabecular bone in SAMP6 than SAMR1. Scale bar, 100 µm. (D) TRAP staining of femur sections indicated more osteoclasts in the BM of SAMP6 than SAMR1. Scale bar, 20 µm. Number of TRAP^+^ osteoclasts in distal marrow per tissue areas (n = 5). (E) WB analysis showed elevated p21, p16 and p53 of BMSCs isolated from 3‐mon SAMP6 than SAMR1. (F) *β*‐gal staining indicated higher BMSCs senescence isolated from 3‐mon SAMP6 than that of SAMR1. Scale bar, 100 µm. (G) ALP staining indicated impaired osteogenesis of BMSCs isolated from 3‐mon SAMP6 than SAMR1. (H) ARS staining indicated impaired osteogenesis of BMSCs isolated from 3‐mon SAMP6 than SAMR1. (I) Immunohistochemical staining indicated higher expression of NETs‐markers MPO and NE in the BM of 3‐mon SAMP6 than SAMR1. Scale bar of 20 µm. (J) Immunofluorescence staining indicated more citH3^+^Ly6G^+^ BM cells in BM of 3‐mon SAMR1 and SAMP6. Scale bar of 20 µm. (K) Immunofluorescence of NETs (DNA and MPO) under PMA stimulation in vitro indicated BM neutrophils of 3‐mon SAMP6 showed more NETs release than SAMR1. Scale bar of 50 µm. n = 5 biologically independent samples. (L) Immunofluorescence indicated BM neutrophils of 3‐mon SAMP6 showed the highest NETosis compared with BM neutrophils of 8‐week and 6‐mon SAMP6 under PMA stimulation in vitro. Scale bar of 20 µm. (M) Immunofluorescence indicated BM neutrophils of 9‐mon male C57BL6/J mice showed the highest NETosis compared with BM neutrophils of 8‐week and 18‐mon mice under PMA stimulation in vitro. Scale bar of 50 µm. (N) WB analysis indicated NETs activated p21, p16 expression of BMSCs and DNase I stimulation attenuated their effects. (O) *β*‐gal staining indicated NETs induced BMSCs senescence in vitro, and DNase I largely abrogated the effects. Scale bar, 100 µm. n = 5 biologically independent samples. (P) Immunofluorescence showed NETs induced ROS production of BMSCs while reversed by DNase I administration. Scale bar,200 µm. Data are represented as mean ± SD. *p < 0.05, **p < 0.01, ***p < 0.001, ****p < 0.0001. Data were analyzed by two‐tailed Student t‐test.

To determine the initial stage of bone aging for SAMP6 mice, we investigated femur bone microstructure of 8‐week and 3‐mon male SAMP6 mice, and we found the latter showed significant bone loss than their age‐matched SAMR1 mice. For 3‐mon male SAMP6 and SAMR1, micro‐computed tomography (µCT) analysis of the distal femur metaphysis showed significantly reduced bone mineral density (BMD), bone volume/tissue volume ratio (BV/TV), trabecular number (Tb. N) and trabecular thickness (Tb. Th) in SAMP6 than SAMR1 (Figure 1A,B), while trabecular separation (Tb. Sp) significantly increased in SAMP6 (Figure 1B). H&E staining confirmed reduced trabecular bone (Figure 1C), and TRAP staining indicated a modest increase in osteoclast numbers in 3‐mon SAMP6 (Figure 1D). In contrast, we found the femur bone mass of 8‐week male SAMP6 was of no significant difference with 8‐week male SAMR1 (Figure S1A, B). Therefore, we identified 3 months of age as the early stage of bone aging in male SAMP6 mice.

Further, we probed BMSCs senescence in the femoral BM of 3‐mon SAMP6 as another hallmark of bone aging. First, immunofluorescence analysis showed up‐regulated γH2A.X expression in the BM of 3‐mon male SAMP6 than SAMR1 (Figure S1C), indicating DNA damage and cellular senescence. Then we isolated mice femoral BMSCs, and western blot analysis showed elevated senescence‐related protein p16, p21 and p53 in BMSCs of SAMP6 than those of SAMR1 (Figure 1E; quantification analysis shown in Figure S1D). Consistently, *β*‐galactosidase (*β*‐gal) staining revealed enhanced senescence in BMSCs of SAMP6 than SAMR1 (Figure 1F; quantification analysis shown in Figure S1E). Moreover, after osteogenic induction of BMSCs for respectively 7 and 14 days, both ALP and ARS staining indicated impaired osteogenic potential of BMSCs in SAMP6 than SAMR1 (Figure 1G,H), consistent with reduced mRNA levels of *Runx2*, *Bglap* and *Sp7* (Figure S1F). These results indicate significant BMSCs senescence and inhibited osteogenesis in 3‐mon male SAMP6 mice.

To compare with physiological aging, we also investigated the early stage of bone aging in C57BL6/J mice. While 18‐to‐24‐mon old are usually considered as the late stage of aging in this mice strain [18], we found male C57BL6/J mice began to show significant bone loss at 9‐mon old than 8‐week mice. µCT, H&E analysis confirmed significant bone loss in 9‐mon male mice than 8‐week male mice (Figure S1G‐I). Also, BMSCs senescence was more evident in 9‐mon C57BL6/J mice BM with higher expression of γH2A.X (Figure S1J) and enhanced *β*‐gal staining in isolated BMSCs (Figure S1K). Together, these results indicate that bone aging in male C57BL/6J mice begins as early as 9 months of age.

### 2.2 Neutrophils of 3‐mon Male SAMP6 Exhibit Activated NETosis

As 3‐mon male SAMP6 mice have shown bone loss compared with SAMR1, we examined neutrophils NETosis within their femoral BM. First, immunohistochemistry and immunofluorescence indicated higher expression of NETosis‐related proteins including MPO, NE, citrullinated histone 3(citH3) in the BM of 3‐mon male SAMP6 than those in age‐ and gender‐matched SAMR1 (Figure 1I,J; quantification analysis shown in Figure S1L). Then, we isolated BM neutrophils through density gradient centrifugation and flow sorting of Ly6G^+^CD11b^+^ cells (Figure S1M). Upon PMA stimulation in vitro, immunofluorescence demonstrated BM neutrophils of 3‐mon SAMP6 released more NETs than SAMR1 controls (Figure 1K; quantification analysis shown in Figure S1L). Overall, these results indicated activated NETosis of BM neutrophils in 3‐mon male SAMP6, concomitant with the onset of bone aging.

We next investigated the temporal dynamics of NETosis activity in BM neutrophils during SAMP6 mice aging. Interestingly, we found BM neutrophils isolated from 3‐mon male SAMP6 showed the highest NETosis than those from 8‐week and 6‐mon mice under PMA stimulation in vitro (Figure 1L; quantification analysis shown in Figure S1N). These results suggest that NETosis of BM neutrophils was activated during the early aging stage of SAMP6 but inactivated at later stages.

Similarly, in C57BL6/J mice, we found higher MPO, NE and citH3 expression in the BM of 9‐mon mice compared with 8‐week controls (Figure S2A, B), consistent with results in SAMP6. Also, flow‐sorted Ly6G^+^CD11b^+^ neutrophils from BM of 9‐mon male mice (Figure S2C) showed activated NETosis under PMA stimulation compared with BM neutrophils from 8‐week and 18‐mon mice (Figure 1M). Collectively, these results confirm NETosis activation in BM neutrophils occurs at the early stage of bone aging.

### 2.3 NETs Induce BMSCs Senescence and Impair Osteogenesis In Vitro

NETs have been reported to be detrimental to tissue cells by inducing cellular damage [19]. To investigate how activated neutrophil NETosis impacts BM cells, we studied their effects on BMSCs senescence through co‐culture in vitro. We isolated BM neutrophils of 3‐mon male SAMP6, collected their NETs and then established NETs‐BMSCs co‐culture model for 48 h. As NETs are mainly composed of dsDNA, we also investigated whether DNA degradation in NETs via DNase I administration could attenuate their effects. Western blot analysis revealed that NETs upregulated p16 and p21 expression of BMSCs, while DNase I largely abolished these effects (Figure 1N; quantification shown in Figure S2D). Consistently, *β*‐gal staining showed that NETs induced senescence in BMSCs, which was largely abrogated by DNase I treatment (Figure 1O; quantification shown in Figure S2E). Meanwhile, NETs activated ROS expression in BMSCs, but the effects were attenuated by DNase I (Figure 1P).

48 h after NETs stimulation in vitro, we conducted osteogenic induction of BMSCs for 7 days or 14 days. ALP and ARS staining manifested NETs stimulation blunt osteogenic differentiation of BMSCs, while DNase I treatment largely rescued these effects (Figure S2F), consistent with qRT‐PCR results showing reduced expression of *Runx2, Bglap* and *Sp7* (Figure S2G).

On the other hand, we collected NETs from BM neutrophils of 9‐mon male C57BL6/J mice, and we found their NETs significantly induced senescence and ROS production in BMSCs isolated from 8‐week male C57BL6/J mice (Figure S2H, I). Consistently, osteogenesis of BMSCs was significantly inhibited by NETs stimulation, as indicated by ALP and ARS staining (Figure S2J).

Mechanistically, we found NETs activated cGAS‐STING, cellular sensor of extracellular DNA in BMSCs (Figure S2L), while inhibiting STING through siRNA transfection (Figure S2K) significantly rescued NETs‐induced cellular senescence, indicated by WB and *β*‐gal analysis (Figure S2K–N). These results indicated extracellular DNA within NETs as important senescence‐inducers.

### 2.4 Early NETs‐Clearance Ameliorates Bone Aging in 3‐mon Male SAMP6

**FIGURE 2:**
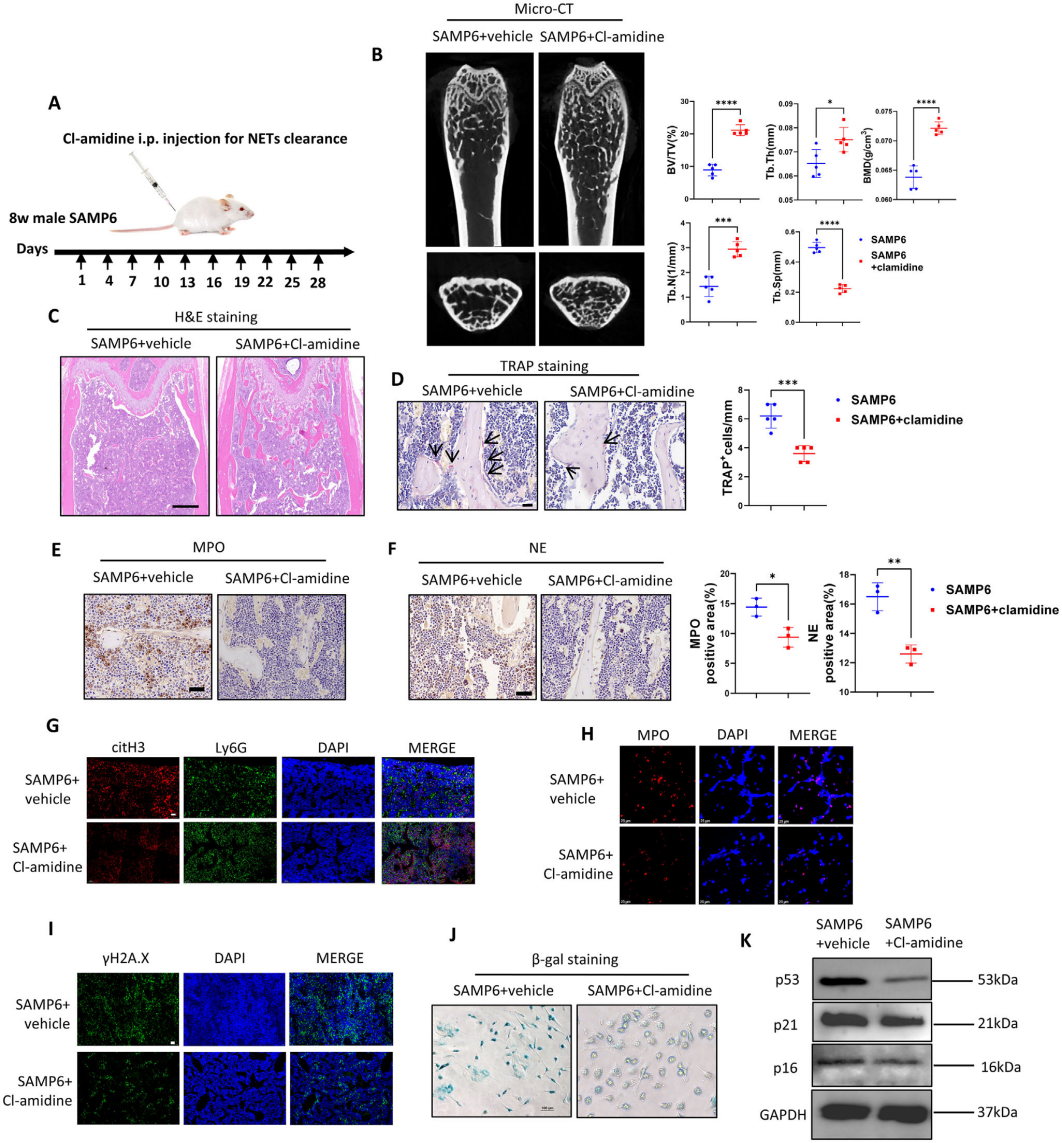
NETs‐clearance delayed bone aging of 3‐mon SAMP6. (A) Scheme of in vivo NETs‐clearance through Cl‐amidine‐injection. (B) µCT indicated NETs‐clearance through Cl‐amidine significantly rescued femur bone loss of 3‐mon SAMP6. Quantitative µCT analysis of distal end of femurs indicated Cl‐amidine administration significantly increased BMD, BV/TV, Tb.th, Tb. N and decreased Tb. Sp of SAMP6. n = 5 mice per group. (C) H&E analysis from the femur metaphysic indicated increased trabecular bone in SAMP6 after Cl‐amidine administration. Scale bar, 200 µm. (D) TRAP staining indicated Cl‐amidine reduced osteoclast number in SAMP6 BM. Scale bar, 20 µm. n = 5 biologically independent samples. (E, F) Immunohistochemical staining indicated Cl‐amidine significantly decreased MPO and NE in the BM of SAMP6, with scale bar of 50 µm. n = 3 biologically independent samples. (G) Immunofluorescence staining of citH3 and Ly6G showed Cl‐amidine significantly decreased citH3 in Ly6G^+^ BM neutrophils. Scale bar of 50 µm. n = 5 biologically independent samples. (H) Immunofluorescence indicated BM neutrophils isolated from Cl‐amidine‐recipient showed significantly inhibited NETosis under PMA stimulation in vitro. n = 5 biologically independent samples. Scale bar, 50 µm. (I) Immunofluorescence staining showed decreased γH2A.X expression in BM of Cl‐amidine‐recipient. Scale bar, 50 µm. (J) *β*‐gal staining indicated BMSCs isolated from Cl‐amidine‐recipient showed lower senescence. Scale bar, 100 µm. (K) WB indicated BMSCs isolated from Cl‐amidine‐recipients showed decreased p53, p21 and p16 expression than BMSCs of SAMP6. Data were represented as mean ± SD. Data were analyzed by two‐tailed Student t‐test. *p < 0.05, **p < 0.01, ***p < 0.001, ****p < 0.0001.

As we identified activated NETosis of BM neutrophils concurrent with the onset of bone aging in 3‐mon male SAMP6, we next investigated whether NETs‐clearance could attenuate bone aging. Male SAMP6 mice received *i.p*. injections of PADI4 inhibitor Cl‐amidine starting at 8 weeks of age for 4 consecutive weeks to deplete NETs, and femurs were harvested at 3 months of age (Figure 2A).

As a result of NETs‐depletion, we found bone aging of 3‐mon male SAMP6 was significantly ameliorated. µCT analysis showed significantly increased BMD, BV/TV, Tb. N and Tb. Th along with decreased Tb. Sp in distal femur metaphysis of Cl‐amidine‐treated mice compared with 3‐mon SAMP6 (Figure 2B). H&E staining further confirmed the rescued bone mass of SAMP6 after NETs clearance (Figure 2C). TRAP staining indicated NETs‐clearance decreased osteoclasts in BM of SAMP6 (Figure 2D). These results indicated NETs‐clearance effectively mitigated bone loss of 3‐mon male SAMP6.

Further, immunostaining and immunofluorescence analysis indicated significantly decreased MPO, NE and citH3 expression in Cl‐amidine‐recipients than SAMP6 controls (Figure 2E–G; quantification shown in Figure S3A). Meanwhile, BM neutrophils isolated from femurs of Cl‐amidine‐recipient mice showed impaired NETosis than 3‐mon SAMP6 under PMA stimulation in vitro (Figure 2H; Figure S3B). These results confirmed NETs‐inhibitory effects of Cl‐amidine.

Then we investigated effects of in vivo Cl‐amidine‐treatment on BMSCs, and we found NETs‐depletion rescued BMSCs senescence of 3‐mon SAMP6. First, immunofluorescence staining for γH2A.X showed a markedly decrease of senescent cells in the BM of Cl‐amidine‐treated mice (Figure 2I; quantification shown in Figure S3C). Then we isolated mice femur BMSCs, and *β*‐gal staining consistently revealed attenuated BMSCs senescence in Cl‐amidine‐recipients (Figure 2J; quantification shown in Figure S3D), accompanied by decreased p16, p21 and p53 expression shown by WB analysis (Figure 2K, quantification shown in Figure S3E). Also, after osteogenic induction, we found mRNA levels of *Runx2*, *Bglap* and *Sp7* significantly increased in BMSCs of Cl‐amidine‐recipient than untreated SAMP6 (Figure S3F).

Taken together, these results indicated bone aging of 3‐mon SAMP6 was significantly rescued by early NETs‐clearance in vivo, underscoring the driving role of neutrophil NETosis in bone aging initiation.

### 2.5 scRNA‐seq Indicates that Neutrophil Subset C10 Accumulates in 3‐mon Male SAMP6 BM With Upregulated CD55 Expression and Activated NETosis

Given the well‐recognized heterogeneity of bone marrow neutrophils, we conducted single cell RNA sequencing (scRNA‐seq) to specify the neutrophil subset most prone to NETosis.

**FIGURE 3:**
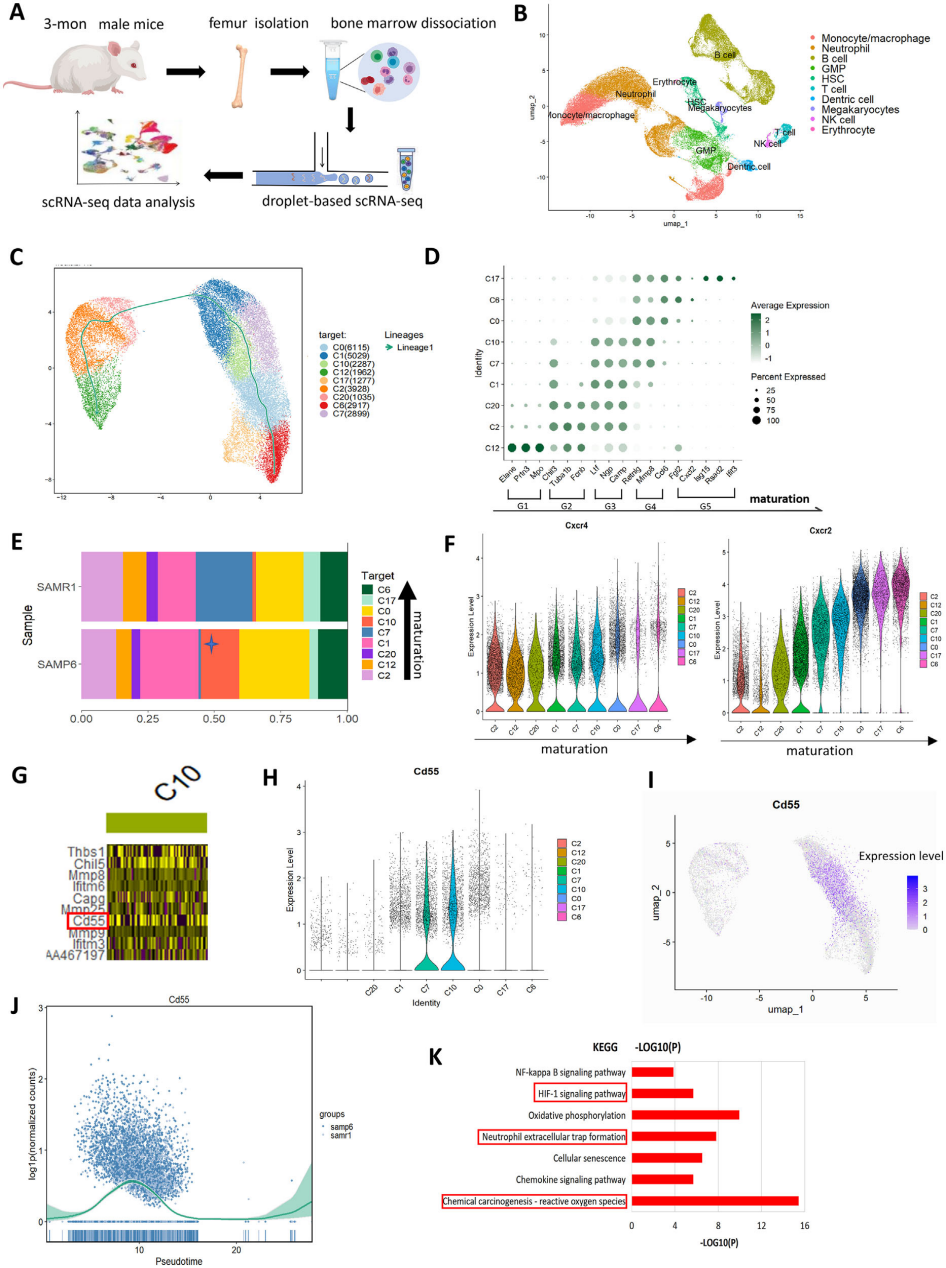
scRNA‐seq analysis of BM neutrophils of 3‐mon male SAMP6 and SAMR1. (A) Flowchart of scRNA‐seq analysis of BM cells of 3‐mon male SAMP6 and SAMR1. (B) UMAP profile indicated cell clusters of BM cells of SAMP6 and SAMR1. (C) UMAP profile indicated SAMP6 and SAMR1 BM neutrophils contained 9 subsets. Pseudo‐time analysis (green curve) of neutrophils subsets. (D) Analysis of neutrophil differentiation–related genes in 9 subsets, and C12 was identified as the least mature and C17 as the most mature, consistent with differentiation trajectory of pseudo‐time analysis. (E) Proportion analysis of neutrophil subsets in SAMP6 and SAMR1 indicated C10 accumulation in SAMP6. (F) Violin plot showed the CXCR4 and CXCR2 expression in neutrophil subsets along with neutrophil maturation. High expression of CXCR4 and intermediate expression of CXCR2 was shown in C10. (G) Heatmap of gene expression showed CD55 significantly upregulated in C10. (H) Violin plot showed the highest expression of CD55 in C10. (I) Projection of CD55‐expression in SAMP6 BM neutrophils showed its expression concentrated in C10. (J) Pseudo‐time analysis of CD55 expression trajectory indicated it exclusively expressed in the middle of neutrophil differentiation. (K) KEGG analysis indicated NETs‐formation, ROS‐related pathway and HIF1‐related pathway significantly activated in neutrophil subset C10.

We isolated femur BM cells from 3‐mon male SAMP6 and SAMR1 for scRNA‐seq analysis (Figure 3A). We merged data of SAMR1 and SAMP6, and after quality control through Cell Ranger and Seurat package (Figure S4A‐C), we obtained 57312 cells with an average of about 1000 feature_RNAs each cell. Further, using Uniform Manifold Approximation and Projection (UMAP), we identified 10 major clusters of bone marrow (BM) cells (Figure 3B), which were annotated based on canonical cell‐type‐specific markers [20, 21] of B lymphocyte (*Cd79a, Cd19, Cd79b*), neutrophils (*Ly6g*, *Cd177*), dendritic cells (*Siglech*), granulocyte‐macrophage progenitor cells (GMPs) (*Mpo, Prtn3*), monocyte/macrophage (*Csf1r*), hematopoietic stem and progenitor cells (HSCs) (*Kit*), T lymphocyte (*Cd3e*), NK cell (*Klrb1c*), erythrocyte (*Hbb‐bt*), and megakaryocytes (*Ms4a2*) (Figure S5A‐C).

Further we focused on scRNA‐seq data of the BM neutrophils subset. Neutrophils respectively made up for 47.4% and 48.2% of SAMR1 and SAMP6 BM cells, and subcluster analysis revealed 9 neutrophil subsets (Figure 3C; Figure S6A). Through pseudo‐time analysis by Slingshot, we investigated neutrophil differentiation trajectory (Figure 3C, the green curve). To define the maturation direction of the pseudo‐time analysis, we examined established neutrophil markers from previous studies [6, 22] in the 9 subsets. We found C12 to be the least and C6/C17 as the most mature subsets, consistent with pseudo‐time analysis (Figure 3D). According to the classification of Xie et al. [6], G1 corresponds to pro‐Neus (progenitor neutrophil), G2 to pre‐neutrophils (preNeu), G3 to immature neutrophils (immNeu) and G4‐5 to mature neutrophils (mNeu). Accordingly, for the 9 subsets, C12 was of stage G1, C2 and C20 of G2‐G3, C1, C7 and C10 of G3‐G4, while C0, C6 and C17 of stage G4‐G5.

Next, we investigated the proportion of the 9 neutrophil subsets in the BM of SAMP6 and SAMR1. Projection of pseudo‐time analysis with data of SAMP6 and SAMR1(Figure S6B), and bar plot analysis (Figure 3E) showed these two samples contained different proportions of neutrophil subsets. Notably, C10 subset significantly accumulated in SAMP6 (14.5% of neutrophils) compared with SAMR1(1.48%) (Figure 3E; Table S1). As for neutrophil differentiation stage based on pseudo‐time analysis, C10 was in stage of G3 to G4 (Figure 3D), a stage between immature and mature neutrophils. CXCR4 and CXCR2 have been recognized as crucial markers of neutrophil differentiation stages, with CXCR4^hi^CXCR2^lo^ indicating immature and intermediate mature neutrophils while CXCR4^lo^CXCR2^hi^ indicating mature neutrophils. Consistently, violin plot (Figure 3F) and feature plot (Figure S6C) indicated high expression of CXCR4 and intermediate expression of CXCR2 in C10. Therefore, we identified C10 as intermediate mature neutrophils. For additional markers indicating neutrophil maturation and differentiation stages, we found high expression of Ly6G, low expression of C/EBPɛ and minimal expression of cKit in C10 (Figure S6D), further confirming C10 as intermediate mature neutrophils.

Then we analyzed gene expression of C10. Heat map revealed the top 10 differentially expressed genes (DEGs) across the 9 neutrophil subsets (Figure S6E), among which CD55 was significantly upregulated in C10 (Figure 3G). Consistently, violin and feature plot both indicated CD55‐expressing cells of SAMP6 mainly concentrated in C10 (Figure 3H, I). Also, pseudo‐time analysis demonstrated that CD55 exclusively peaked in the middle stage of neutrophil differentiation (Figure 3J), consistent with C10 maturation stage. Although C7 cells also exhibited some CD55 expression (Figure 3H), their expression was still much lower than C10 (Figure S6F). Therefore, we identified CD55 as a specific marker of neutrophil subset C10 in SAMP6.

Finally, to investigate functions of C10, we performed KEGG analysis and found significant enrichment of NETs‐formation, ROS‐related pathways and HIF‐1 related pathway (Figure 3K; Figure S6G). GO (gene ontology) analysis for CD55‐related pathway in C10 indicated upregulation of mitosis, cell cycle and cell differentiation related process (Figure S6H). These results implied C10 as a crucial neutrophil subset with activated NETosis in 3‐mon male SAMP6 BM, and CD55 serving as its specific marker.

### 2.6 CD55^+^Ly6G^+^CD11b^+^ Represents the Intermediate Mature Neutrophil Subset Accumulated in 3‐mon SAMP6 BM

To investigate whether CD55^+^Ly6G^+^CD11b^+^ neutrophils correspond to the neutrophil subset C10 accumulating in 3‐mon SAMP6 BM, we examined their abundance and their differentiation stage in the BM of SAMP6 and SAMR1 mice.

**FIGURE 4:**
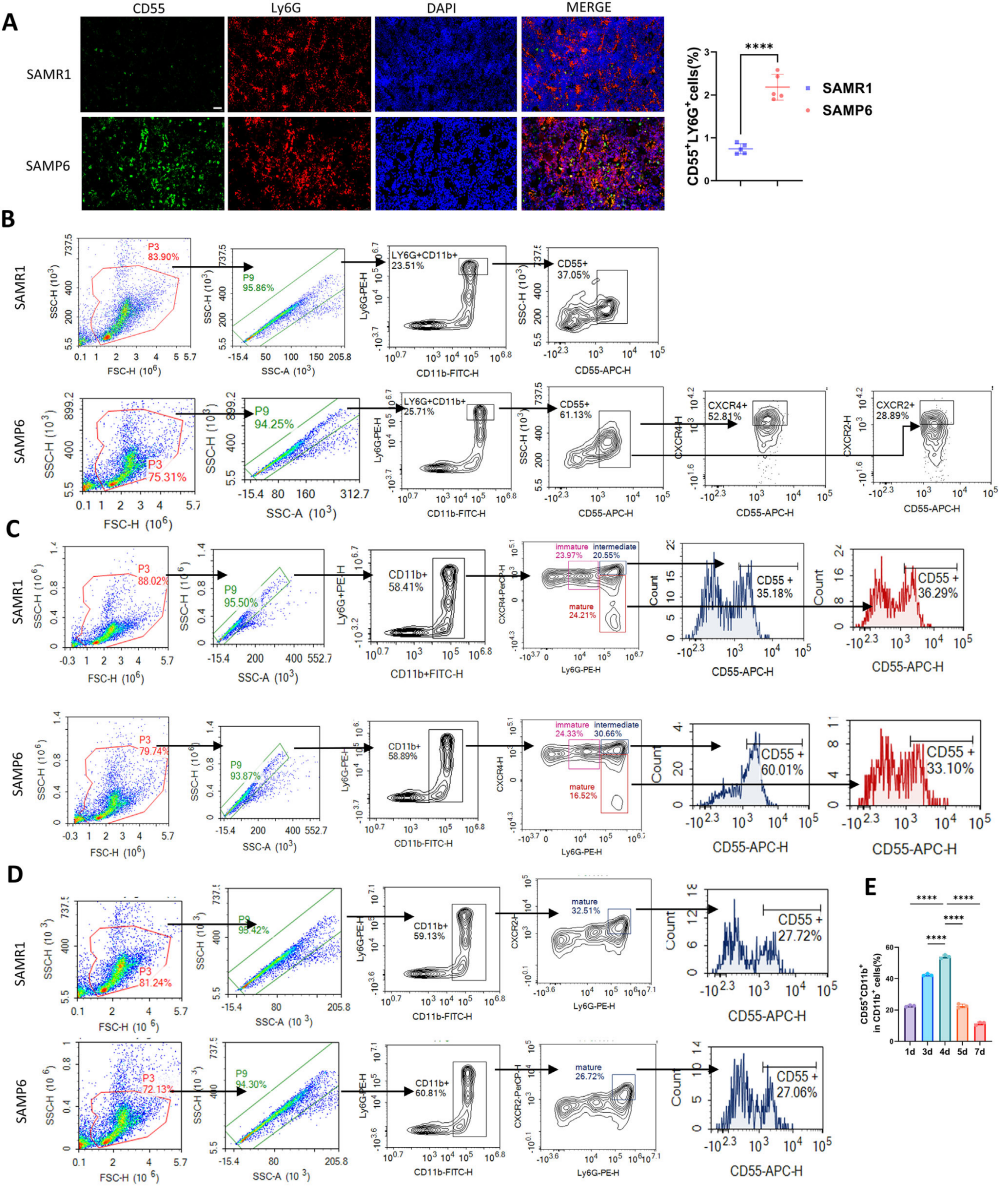
CD55^+^Ly6G^+^CD11b^+^ intermediate mature accumulated in 3‐mon SAMP6. (A) Immunofluorescence indicated 3‐mon SAMP6 contained more CD55^+^Ly6G^+^ cells in BM than SAMR1. Scale bar, 50 µm. n = 5 biologically independent samples. (B) Flow cytometry analysis indicated 3‐mon SAMP6 BM contained more CD55^+^Ly6G^+^CD11b^+^ neutrophils than SAMR1, and CD55 showed high co‐expression with CXCR4 while low co‐expression with CXCR2. (C) Flow cytometry indicated 3‐mon SAMP6 with higher amount of intermediate mature neutrophils and lower mature neutrophils than SAMR1. CD55 expression in intermediate mature neutrophils of SAMP6 was higher than SAMR1, while CD55 was of similarly low level in mature neutrophils of both SAMP6 and SAMR1. (D) CXCR2^+^Ly6G^+^CD11b^+^ (mature neutrophils) decreased in BM of 3‐mon SAMP6 than SAMR1. CD55 showed low expression in mature neutrophils of both SAMP6 and SAMR1. (E) CD55 exhibited peak expression at the intermediate stage of HL‐60 cell differentiation into mature neutrophils. Data were represented as mean ± SD. Data were analyzed by two‐tailed Student t‐test. ****p < 0.0001.

First, we verified the accumulation of CD55^+^ neutrophils in 3‐mon male SAMP6 BM. Immunofluorescence staining indicated up‐regulated co‐localization of CD55 with Ly6G^+^ BM neutrophils in 3‐mon SAMP6 BM than SAMR1 (Figure 4A). Also, flow cytometry indicated increased CD55 expression in Ly6G^+^CD11b^+^ BM neutrophils of 3‐mon male SAMP6 than SAMR1 (Figure 4B, quantified in Figure S7A). These results confirmed enriched CD55^+^Ly6G^+^CD11b^+^ neutrophils in 3‐mon SAMP6 BM. Similarly, we found significant accumulation of CD55^+^ neutrophils in 9‐mon male C57BL6/J mice BM compared with 8‐week controls (Figure S7B, C).

Further, we investigated maturation stage of CD55^+^Ly6G^+^CD11b^+^ neutrophils. Previous studies have defined Ly6G^lo^CXCR4^hi^ neutrophils as immature neutrophils, Ly6G^hi^CXCR4^hi^ as intermediate mature neutrophils and Ly6G^hi^CXCR4^lo^CXCR2^+^ as mature neutrophils, all of which show high CD11b expression [6, 23]. Therefore, we examined CXCR4 and CXCR2 expressions in CD55^+^Ly6G^+^CD11b^+^ neutrophils. We found Ly6G^+^CD11b^+^CD55^+^ cells of SAMP6 mice showed high CXCR4 co‐expression (52.81%) while low CXCR2 expression (28.89%) (Figure 4B; quantified in Figure S7A). Comparable results of CXCR4 and CXCR2 expression were observed in Ly6G^+^CD11b^+^CD55^+^ cells of SAMR1 (Figure S7D). Consistently, we found CD55^+^ neutrophils of 9‐mon male C57BL6/J mice also showed high CXCR4 expression and low CXCR2 expression (Figure S7B), indicating conserved intermediate‐mature characteristics of BM CD55^+^ neutrophils across different murine strains.

Flow cytometry also revealed the proportions of neutrophils of different maturation stages in the BM of SAMP6 and SAMR1 mice. First, we found intermediate mature neutrophils (CD11b^+^Ly6G^hi^CXCR4^hi^) enriched while mature neutrophils (CD11b^+^Ly6G^hi^CXCR4^lo^) decreased in BM of SAMP6 compared with SAMR1 (Figure 4C; quantified in Figure S7E). Similarly, CD11b^+^Ly6G^hi^CXCR2^+^ mature neutrophils decreased in BM of 3‐mon male SAMP6 compared with SAMR1 mice (Figure 4D; quantified in Figure S7F). Further, within the BM intermediate mature neutrophils, CD55^+^ cells significantly increased in 3‐mon SAMP6 than SAMR1 (Figure 4C; quantified in Figure S7E). However, in CD11b^+^Ly6G^hi^CXCR2^+^ mature neutrophils, CD55 expression was consistently low in both SAMP6 and SAMR1 groups (Figure 4C,D; quantified in Figure S7F). These results resonated with scRNA‐seq results, supporting that CD55^+^Ly6G^+^CD11b^+^ neutrophils were at the intermediate mature stage.

Meanwhile, we investigated the expression pattern of CD55 during neutrophil differentiation. We induced neutrophil‐like granulocyte differentiation of HL‐60 cells (human acute promyelocytic leukemia cell line) using administration of 1.25% DMSO in vitro. Flow cytometry revealed CD55 expression peaked in the middle (day 4) as CD11b expression gradually increased during neutrophil maturation from day 1 to day 5 through flow cytometry (Figure 4E; Figure S7G). These above results identified CD55 as a characteristic marker of the intermediately mature neutrophils C10 that accumulated in the BM of 3‐mon male SAMP6.

### 2.7 CD55^+^ Neutrophils Showed Up‐Regulated NETosis via CD55‐HIF1α‐PADI4 Pathway

**FIGURE 5:**
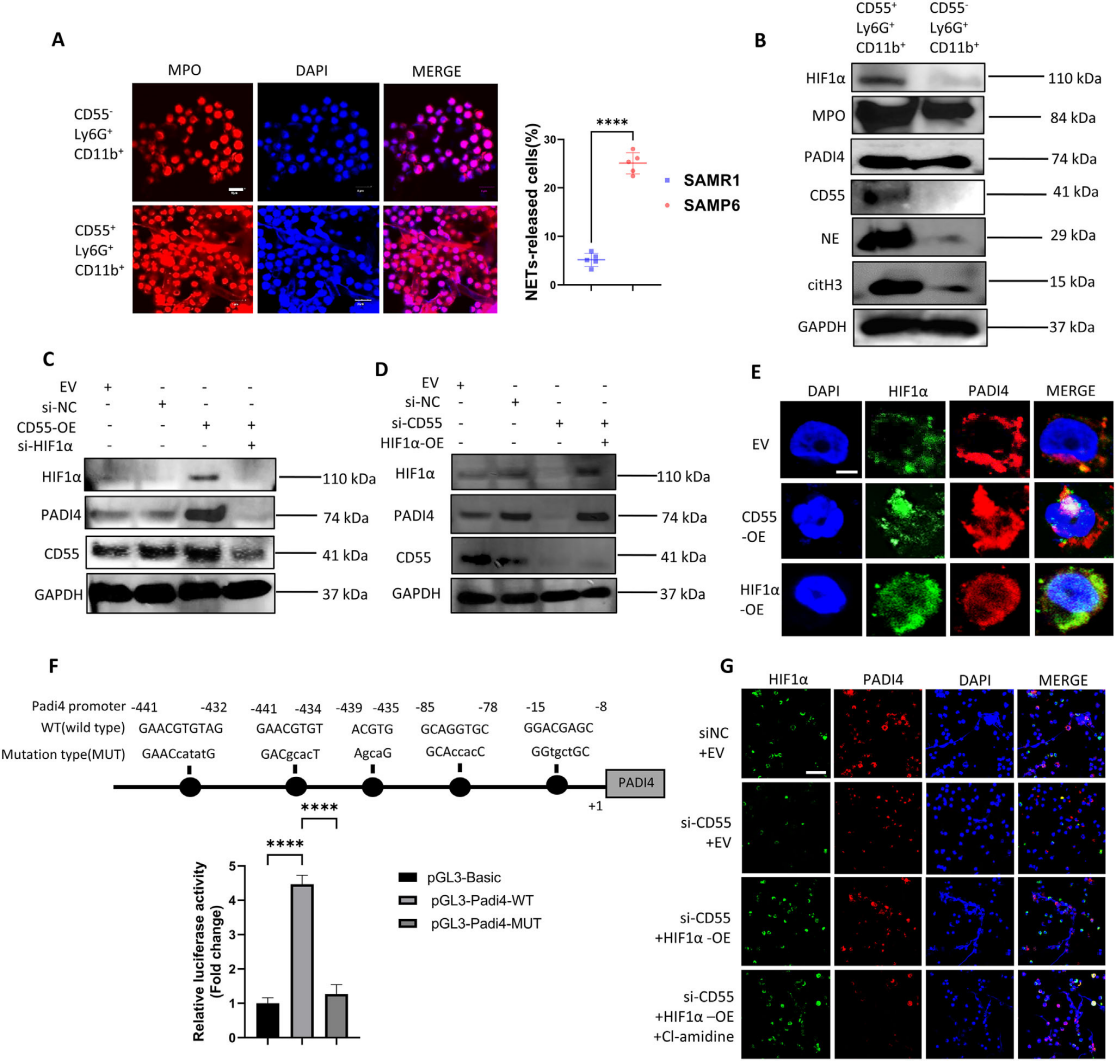
CD55^+^Ly6G^+^CD11b^+^ neutrophils showed activated NETosis through mediation of CD55‐HIF1α‐PADI4 pathway. (A) Immunofluorescence showed CD55^+^ neutrophils isolated from BM of 3‐mon male SAMP6 released more NETs than CD55^−^ neutrophils in vitro under H_2_O_2_ stimulation. Scale bar, 5 µm. n = 5 biologically independent samples. (B) WB analysis indicated higher expression of HIF1α, MPO, PADI4, NE and citH3 in CD55^+^ neutrophils than CD55^−^ neutrophils. (C) CD55‐OE up‐regulated HIF1α and PADI4 expression of HL‐60, while was further abrogated by transfection of HIF1α‐siRNA. (D) CD55 knockdown by siRNA transfection down‐regulated HIF1α and PADI4 expression of HL‐60, which was further rescued by transfection of HIF1α‐OE plasmid. (E) CD55‐OE activated HIF1α and PADI4 expression and nuclear translocation. Scale bar, 2 µm. (F) Dual luciferase reporter gene analysis indicated HIF1α combined with promoter of PADI4 to activate its expression, while failed to promote its expression when the promoter was mutated in posssible binding sites. (G) Under H_2_O_2_ stimulation in vitro, inhibition of CD55 by siRNA inhibited NETosis and HIF1α expression, while addition of HIF1α‐OE rescued NETosis. PADI4 inhibitor Cl‐amidine finally inhibited both effects of CD55 and HIF1α on neutrophil NETosis. Data were represented as mean ± SD. Data were analyzed by two‐tailed Student t‐test. ****p < 0.0001.

Next, to test NETosis capacity of CD55^+^Ly6G^+^CD11b^+^ according to scRNA‐seq results, we flow sorted these neutrophils from 3‐mon male SAMP6 BM and induced their NETosis in vitro. As KEGG analysis indicated ROS‐related pathways were activated in C10 (Figure 3K), we applied H_2_O_2_ instead of PMA as neutrophil NETosis trigger. Immunofluorescence indicated CD55^+^ neutrophils released more NETs than CD55^−^ neutrophils (Figure 5A), and we found significant co‐localization of CD55 and NETs‐released cells (Figure S8A). Consistently, WB results indicated BM CD55^+^ neutrophils expressed higher level of NETs‐related proteins including PADI4, NE, MPO and citH3 than CD55^−^ neutrophils (Figure 5B; quantification shown in Figure S8B). Meanwhile, HIF1α was up‐regulated in CD55^+^ neutrophils, consistent with our scRNA‐seq results (Figure 5B). These results confirmed that they activated NETosis potential of CD55^+^ neutrophils.

Based on these results, we investigated mechanism underlying activated NETosis of CD55^+^ neutrophils. To investigate whether CD55 directly regulated neutrophil NETosis, we induced neutrophil‐like granulocytes differentiation of HL‐60 to conduct knockdown and overexpression assays. As we found CD55 expression peaked at day 4 of neutrophil differentiation induction (Figure 4E), we transfected siRNA and/or plasmids at day 2 for 2 days during DMSO induction. Given that PADI4 is a key regulator of ROS‐activated NETosis [7, 10], we investigated whether CD55 and HIF1α could regulate *Padi4* expression. We transfected HL‐60 with *CD55*‐OE (over expression) plasmid and/or *HIF1α* siRNA for 48 h. Plasmid and siRNA transfection efficiency were verified by qRT‐PCR (Figure S8C). We found CD55‐OE significantly upregulated both HIF1α and PADI4 levels, while HIF1α knockdown markedly suppressed PADI4 expression (Figure 5C; quantification shown in Figure S8D). However, CD55 was also modestly inhibited by *HIF1α* siRNA (Figure 5C).

Conversely, we transfected HL‐60 cells with *HIF1α*‐OE plasmid and/or *CD55* siRNA, and transfection efficiency results were verified by qRT‐PCR (Figure S8C) and GFP fluorescence (Figure S8E). We found CD55‐silencing significantly inhibited HIF1α and PADI4, while HIF1α‐OE significantly rescued PADI4 but not CD55 expression (Figure 5D; quantification shown in Figure S8F). Consistently, through immunofluorescence we found CD55‐OE induced up‐regulation and nuclear translocation of HIF1α and PADI4, and HIF1α‐OE alone also markedly upregulated PADI4 (Figure 5E). Collectively, these findings suggest that CD55 dominantly activates PADI4 expression through HIF1α, while CD55‐inhibition induced by *HIF1α* siRNA indicating potential dual interaction of CD55 and HIF1α.

Next, we investigated whether transcription factor HIF1α could combine with the promoter of *Padi4* gene to activate its expression. Through dual luciferase reporter gene analysis, we found over expression of HIF1α significantly enhanced luciferase activity driven by the wild‐type *Padi4* promoter but failed to do so when the predicted binding site was mutated (MUT) (Figure 5F). These results indicated HIF1α interacted with the predicted site of *Padi4* promoter to activate PADI4 expression.

Finally, we investigated whether neutrophil NETosis was regulated by CD55‐HIF1α‐PADI4 pathway. We stimulated NETosis of HL‐60‐derived neutrophil‐like cells through H_2_O_2_ in vitro. Through immunofluorescence, we found NETosis significantly declined upon CD55‐inhibition, while HIF1α‐OE significantly rescued NETosis. However, effects of CD55 and HIF1α‐OE was abrogated by PADI4 inhibitor Cl‐amidine (Figure 5G). These findings demonstrated CD55 potentiated neutrophil NETosis via HIF1α‐PADI4 pathway.

### 2.8 In Vivo Transfer of CD55^+^ Neutrophils Induces Bone Aging in 3‐mon Male SAMR1

**FIGURE 6:**
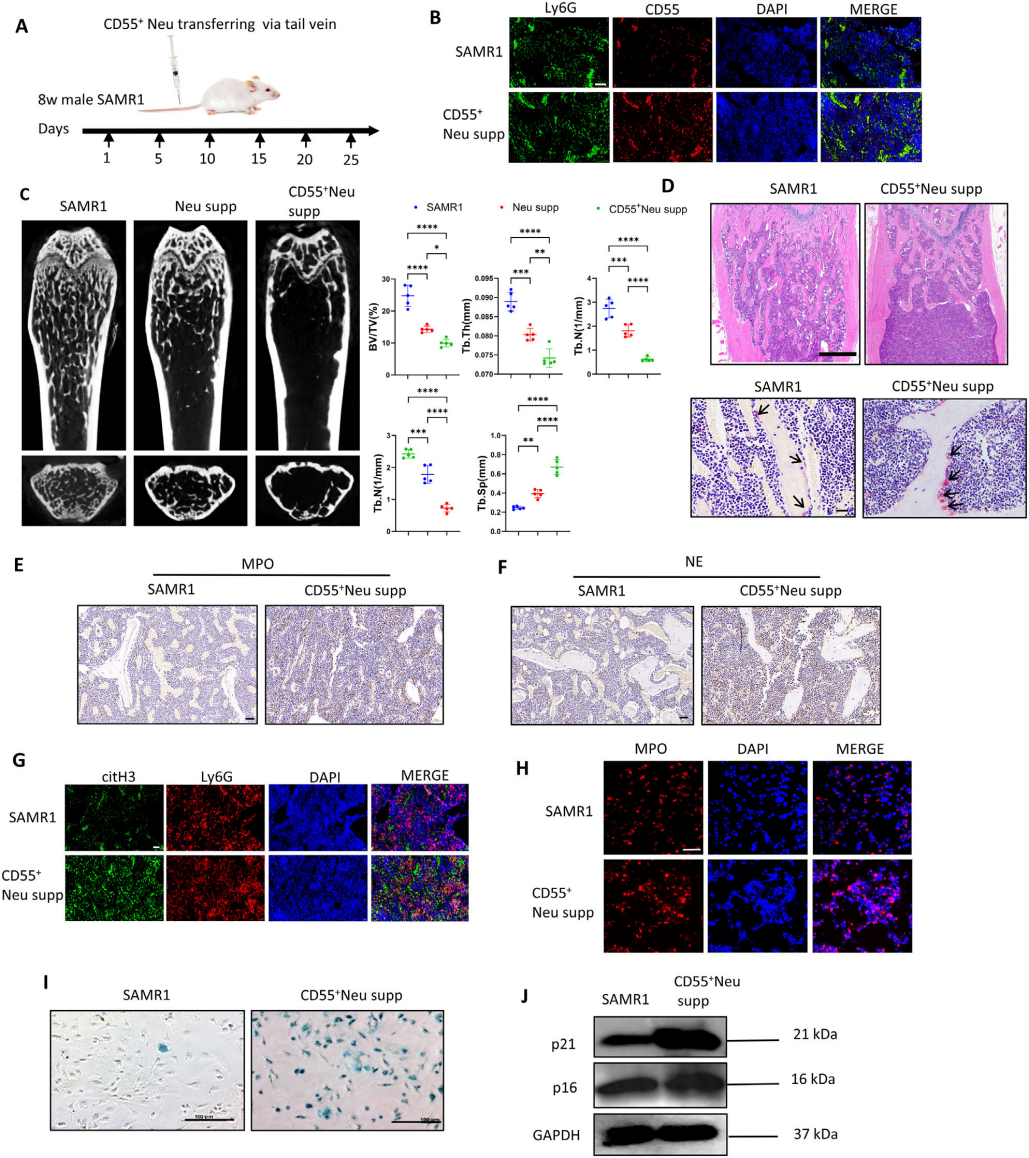
Transferring of CD55^+^ neutrophils to SAMR1 induced bone aging. (A) Scheme of CD55^+^Ly6G^+^CD11b^+^ neutrophils transfer via tail vein to 8‐week SAMR1. (B) Immunofluorescence staining confirmed CD55^+^ neutrophil‐injection significantly increased CD55 expression in Ly6G^+^ BM neutrophils. Scale bar, 50 µm. n = 5 biologically independent samples. Scale bar, 50 µm. (C) µCT analysis indicated transferring of BM neutrophils of SAMP6 or CD55^+^ neutrophils to SAMR1 induced significant femur bone loss, while the later was more significant. Quantitative µCT analysis of distal end of femurs indicated CD55^+^ neutrophil‐injection induced the most significant decrease in BMD, BV/TV, Tb.th, Tb.N and increase in Tb.Sp of SAMR1. n = 5 mice per group. (D) H&E analysis showed significantly reduced trabecular bone in CD55^+^ neutrophils‐recipients than controls. Scale bar, 200 µm. TRAP staining (lower images) indicated increased osteoclast number in BM of CD55^+^ neutrophils‐recipients. Scale bar, 20 µm. n = 5 biologically independent samples. (E, F) MPO and NE were significantly up‐regulated in BM of CD55^+^ neutrophil recipients. Scale bar of 50 µm. n = 3 biologically independent samples. (G) Immunofluorescence staining showed citH3^+^Ly6G^+^ significantly increased in BM of CD55^+^ neutrophil recipients. Scale bar of 50 µm. n = 5 biologically independent samples. (H) BM neutrophils isolated from CD55^+^ neutrophil‐recipients showed significantly increased NETosis than SAMR1 in vitro under H_2_O_2_ stimulation. n = 5 biologically independent samples. (I) *β*‐gal staining indicated BMSCs isolated from CD55^+^ neutrophil‐recipients with higher senescence extent. Scale bar, 100 µm. (J) WB analysis indicated up‐regulated p21 and p16 in BMSCs of CD55^+^ neutrophil‐recipients. Data were represented as mean ± SD. Data were analyzed by One‐way ANOVA with Tukey's post hoc test. *P<0.05, **p < 0.01, ***p < 0.001, ***p < 0.0001.

As we have identified the activated NETosis of BM CD55^+^ neutrophils, we further investigated whether they contributed to bone aging of 3‐mon SAMP6. We sorted CD55^+^Ly6G^+^CD11b^+^ cells from BM of 3‐mon male SAMP6 and then transferred 10^6^ cells/mice each time via tail vein injection into 8‐week male SAMR1 mice every 5 days for consecutive 4 weeks (Figure 6A).

First, we confirmed a marked increase of CD55 expression in Ly6G^+^ BM neutrophils of recipient mice, indicating successful CD55^+^ neutrophils transfer to SAMR1 mice (Figure 6B; quantification shown in Figure S9A). For harvested femur bone of 12‐week‐old male SAMR1, µCT results showed transfer of either total SAMP6 BM neutrophils or purified CD55^+^ neutrophils resulted in marked bone loss, with significantly decreased BV/TV, BMD, Tb. N, Tb. th and increased Tb. Sp than 3‐mon SAMR1 control, with the most pronounced effects observed in the CD55^+^‐neutrophil group (Figure 6C). Consistent with µCT results, in CD55^+^ neutrophils‐recipient group, HE staining showed supplement reduced trabecular bone (Figure 6D) and TRAP staining showed enhanced osteoclast numbers than SAMR1 control mice (Figure 6D; quantification shown in Figure S9B). These results indicated CD55^+^ neutrophils induced significant bone loss.

Further, we investigated NETs protein expression in the BM. Immunohistochemistry and immunofluorescence indicated upregulated NE, MPO and citH3 expression in the BM of CD55^+^ neutrophil‐recipient mice (Figure 6E–G; quantification shown in Figure S9C). Also, BM neutrophils of CD55^+^ neutrophil ‐recipient mice released more NETs than control under H_2_O_2_ stimulation in vitro (Figure 6H; quantification shown in Figure S9D).

Next, we investigated whether CD55^+^ neutrophil supplement also caused BMSCs senescence. We isolated mice femoral BMSCs, and *β*‐gal staining showed increased senescence in BMSCs of CD55^+^ neutrophil‐recipients compared with controls (Figure 6I; quantification shown in Figure S9E). Consistently, WB analysis showed higher expression of p16 and p21 in BMSCs of CD55^+^ neutrophil‐recipients (Figure 6J; quantification shown in Figure S9F). Collectively, these results demonstrated that CD55^+^ neutrophils contributed to bone loss through NETs‐induced BMSCs senescence.

### 2.9 ROS Acts as a Robust In Vivo Inducer of BM Neutrophil NETosis in 3‐mon SAMP6

As scRNA‐seq results revealed elevated ROS‐related activity in BM neutrophils, we investigated whether ROS functions as a NETosis inducer in the BM of SAMP6.

**FIGURE 7:**
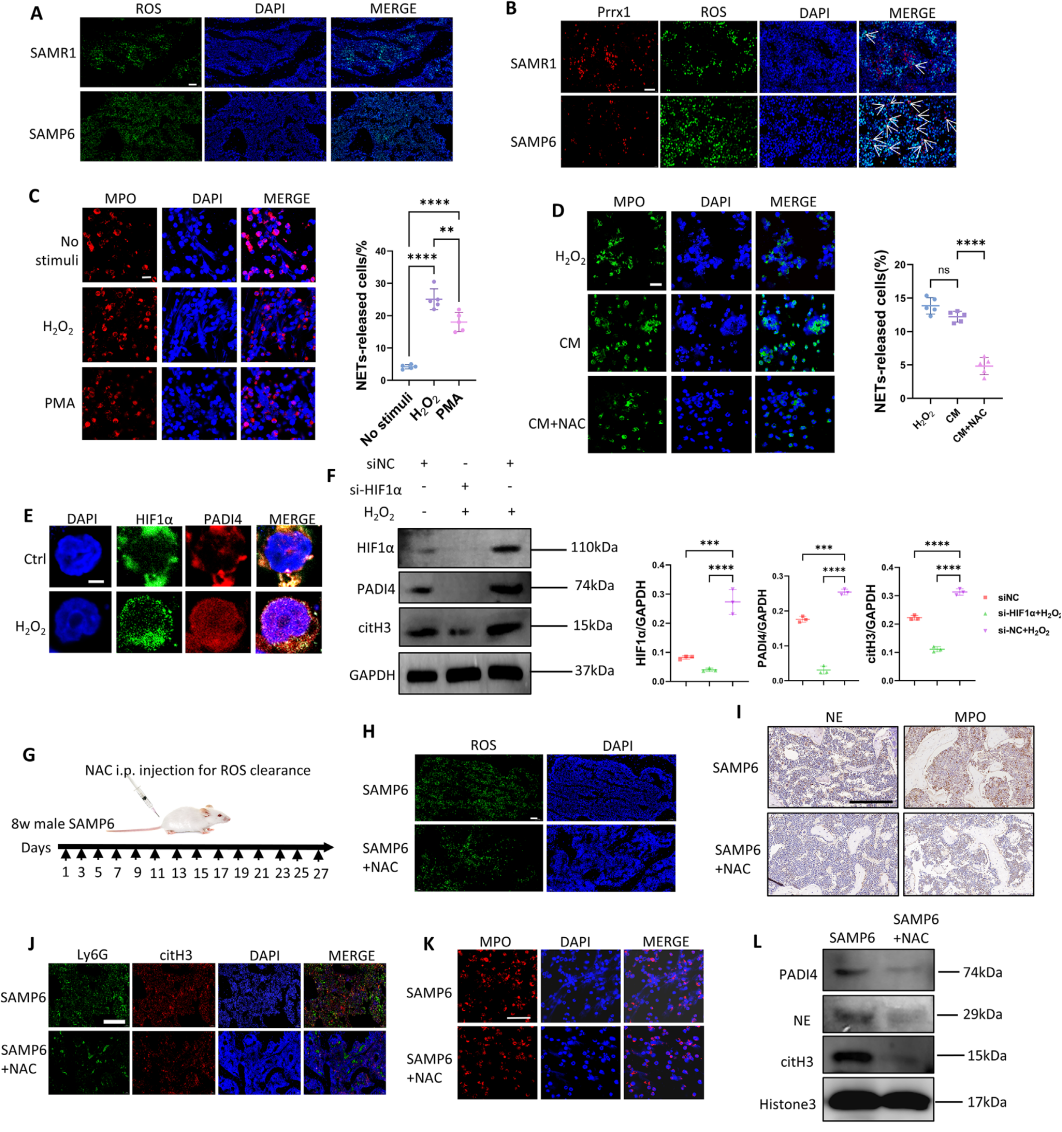
ROS was a robust inducer of neutrophil NETosis in the BM of 3‐mon male SAMP6. (A) Immunofluorescence indicated higher spontaneous ROS levels in the BM of 3‐mon SAMP6 than SAMR1.Scale bar, 20 µm. (B) BMSCs marker Prrx1 showed more significant co‐localization with spontaneous ROS in BM of SAMP6 mice than SAMR1. Arrows refer to double positive cells. (C) NETosis of BM neutrophils of 3‐mon SAMP6 was significantly activated by H_2_O_2_ with similar extent of PMA in vitro. Scale bar, 10 µm. (D) CM of BMSCs of SAMP6 significant induced NETosis of BM neutrophils with similar extent with H_2_O_2_, while administration of NAC abrogated effects of BMSCs CM. (E) H_2_O_2_ induced HIF1α and PADI4 nuclear translocation and up‐regulation. Scale bar, 2 µm. (F) Transfection of HIF1α‐siRNA abrogated H_2_O_2_‐induced up‐regulation of HIF1α, PADI4 and citH3 of HL‐60. (G) Scheme of ROS‐clearance via intraperitoneal injection (i.p.) injection of NAC to 8‐week male SAMP6. (H) Immunofluorescence indicated ROS significantly decreased in BM of NAC‐recipients. Scale bar, 50 µm. (I) MPO and NE significantly decreased in BM of NAC recipients, with scale bar of 100 µm. (J) Immunofluorescence staining indicated citH3 significantly decreased in BM Ly6G^+^ neutrophils of NAC recipients. Scale bar of 50 µm. (K) Immunofluorescence indicated BM neutrophils isolated from NAC recipients released fewer NETs than SAMP6 in vitro under H_2_O_2_ stimulation. Scale bar of 50 µm. (L) WB showed PADI4, NE and citH3 expression of BM neutrophils isolated from NAC recipients was significantly lower than SAMP6 mice. Data were represented as mean ± SD. Data were analyzed by One‐way ANOVA with Tukey's post hoc test. *P<0.05, **p < 0.01, ***p < 0.001, ***p < 0.0001.

First, through immunofluorescence and flow cytometry we found increased ROS levels in BM cells of 3‐mon SAMP6 compared with SAMR1 (Figure 7A; Figure S10A). Consistently, we verified ROS accumulation in the BM of 9‐mon than 8‐week male C57BL6/J mice (Figure S10B). Further, we investigated whether BMSCs produced ROS within BM. We isolated BMSCs from 3‐mon male SAMR1 and SAMP6 mice, and flow cytometry indicated higher ROS expression in the former (Figure S10C). Immunofluorescence further revealed reduced expression of BMSC lineage markers (Prrx1, Sox9, and Runx2) in the BM of 3‐mon male SAMP6 mice, while ROS signals and their co‐localization with these markers were more prominent than in SAMR1 (Figure 7B; Figure S10D). These results suggest BMSCs as a ROS source in SAMP6 BM.

Next, we investigated effects of ROS on neutrophil NETosis and underlying mechanisms. In vitro, we found 200 µM H_2_O_2_ significantly induced NETosis of BM neutrophils, comparable with PMA stimulation and greater than spontaneous NETosis (Figure 7C). Consistently, H_2_O_2_ upregulated NETosis marker PADI4 and MPO expression of BM neutrophils (Figure S10E). NAC(N‐acetylcysteine) increases cellular GSH levels and acts as a direct antioxidant [24]_,_ and we found it effectively abrogated effects of H_2_O_2_ (Figure S10E)_._ As aging bone marrow was sterile micro‐environment in absence of LPS or PMA, these results indicated ROS could serve as a physiological inducer of NETosis in vivo. We also collected conditional medium (CM) of passage 2 BMSCs isolated from 3‐mon male SAMP6 to stimulate neutrophils, and we found the CM similarly triggered neutrophil NETosis in vitro, an effect attenuated by NAC pretreatment (Figure S10D). These results demonstrated the triggering role of BMSCs‐derived ROS in neutrophil NETosis. Previous studies have shown that a high concentration of NAC (15 mM) directly inhibits neutrophil NETosis in vitro by suppressing endogenous ROS [25]. In this study, we used a lower concentration of NAC (200 µM) to primarily scavenge endogenous ROS. Nevertheless, we acknowledged that NAC at this concentration could still exert minor effects on baseline neutrophil NETosis.

To study the mechanism of ROS‐activated neutrophil NETosis, we stimulated HL‐60 at day 4 of neutrophil‐like cell differentiation induction with H_2_O_2_ for 24 h and found H_2_O_2_ induced HIF1α and PADI4 nuclear translocation and upregulation (Figure 7E). Further, we found silencing HIF1α by siRNA transfection for 48 h prior to H_2_O_2_ stimulation significantly abrogated PADI4 activation (Figure 7F). These results indicated H_2_O_2_ could regulate PADI4 through mediation of HIF1α, integrating with CD55‐primed HIF1α‐PADI4 pathway to activate NETosis.

Next, to investigate the effects of ROS on NETosis of CD55^+^neutrophils in vivo, we administered the ROS scavenger NAC intraperitoneally to 8‐week male SAMP6 mice for four consecutive weeks (Figure 7G) and harvest their femurs at 12 weeks. As a result, immunofluorescence confirmed reduced ROS in the BM of NAC‐recipients than untreated SAMP6 controls (Figure 7H; quantification shown in Figure S10F), confirming effective ROS‐clearance. Further, NAC‐recipient mice showed reduced NETosis with lower expression of MPO, NE, citH3 in BM than control SAMP6 mice (Figure 7I,J; quantification shown in Figure S10G). Then we isolated BM neutrophils from SAMP6 controls and NAC‐recipients, and the latter showed significantly fewer NETs released in vitro under H_2_O_2_ stimulation (Figure 7K; quantification shown in Figure S10H) and showed significantly lower PADI4, NE and citH3 expression than SAMP6 (Figure 7L; quantification shown in Figure S10I). Moreover, we found ROS clearance not only inactivated BM neutrophil NETosis but also rescued bone aging (Figure S10J,K). Together, these results validated ROS as a robust in vivo inducer of BM neutrophils NETosis and revealed a new pathway ROS contributed to bone aging.

## 3 Discussion

SAMP6 is an established mice model of spontaneously accelerated aging, and SAMR1 serves as their natural aging control [26]. SAMP6 shows low bone turnover and significant inhibition of bone formation, and has been widely applied for studies of the pathogenesis and treatment study of senile osteoporosis [27, 28]. Although exhibiting premature aging, SAMP6 mice share key mechanisms of bone aging with humans. First, they share the mechanism of BMSCs senescence, characterized by mitochondrial degradation and increased ROS production, leading to significant osteogenesis inhibition [29, 30, 31, 32]. Second, SAMP6 and humans share molecular mechanisms that lead to osteogenesis inhibition, including over‐activation of secreted frizzled‐related protein (Sfrp4) in BMSCs and osteoblasts to inhibit Wnt signaling pathway [33, 34]. Consistent with previous studies [35], our study verified male SAMP6 began to show bone aging as early as 3 months of age with significant bone loss and BMSCs senescence.

Further, for the first time, we found BM neutrophils of 3‐mon SAMP6 manifested activated NETosis, which induced BMSCs senescence and inhibited osteogenesis, leading to initiation of bone aging. Meanwhile, these results were confirmed in C57BL6/J mice. An additional advantage of the SAMP6 model lies in their rapid aging process, as significant bone loss occurs within a short period (from 8 to 12 weeks of age). This feature notably shortens experimental duration, and we found 4 weeks of NETs‐depletion significantly ameliorated bone aging in 3‐mon male SAMP6 mice. In the future, we will continue to assess the optimal timing and duration of NETs depletion and extend our validation to naturally aging mice to further validate translational potential for osteoporosis treatment.

Mechanistically, we found neutrophil heterogeneity and pro‐inflammatory cues collectively contributed to NETosis activation in aging BM. First, we identified an intermediate mature neutrophil subset of CD55^+^Ly6G^+^CD11b^+^ neutrophils in the BM with activated NETosis, and their characteristics and markers are summarized in Table S3. Physiologically, mature neutrophils are thought to have the highest NETosis capacity to combat pathogens [6, 16]. However, during systemic inflammation, immature neutrophils also exhibit significantly enhanced immune response including NETosis [17, 36]. Meanwhile, systemic inflammation triggers emergency neutropoiesis in the BM, characterized by migration of mature neutrophils into the circulation and accelerated differentiation of neutrophil progenitors, leading to increased immature and intermediate mature neutrophils in the BM [22]. Aging has been found to induce systemic inflammation, and Gullotta et al. [16] found activated migration of mature neutrophils from BM to the circulation. Similar with emergency neutropoiesis, our results indicated decreased mature neutrophils while increased number of intermediate mature neutrophils within aging BM. These results suggest that the activation of NETosis in intermediate mature neutrophils rather than mature neutrophils may reflect aging‐associated shifts in neutrophil dynamics.

Further, we uncovered CD55 mediated the activation of neutrophil NETosis through HIF1α‐PADI4 pathway. Previous studies haven't reported the role of CD55 in neutrophil NETosis, but various NETosis triggers including IL‐1*β*, LPS, TNF‐α and fMLP have been found to activate CD55 expression on neutrophils [37, 38]. Also, we found the exclusive expression of CD55 in intermediate mature neutrophils. CD55 was originally a complement inhibitor on cell surface [39], while recent studies have revealed its non‐canonical role of promoting cell proliferation and inhibiting apoptosis [40]. Our results indicated the role of CD55 in neutrophil differentiation and cell cycle, and previous studies have reported the significant cell cycle transition during neutrophils differentiation [23] and the crucial role of CDK4/6‐mediated G1/S phase transition in NETosis induction [41]. These results suggest that CD55 serves as an important mediator linking neutrophil differentiation and NETosis function. CD55 is localized on cell surface within lipid rafts and interact with raft‐resident adaptor proteins to activate downstream pathways including Src family kinases [40]. As Src family kinases have been found to activate HIF1α expression and promote its nuclear translocation [42], we will continue to investigate the precise molecular mechanism of CD55‐dependent HIF1α activation.

Recently, Hsu et al. shed light on the critical role of CD55 in neutrophil function [38]. Consistent with our study, Hsu et al. found CD55 expression on cell surface significantly decreased during neutrophil aging. Meanwhile, they found aged neutrophils secreted CD55^+^ LAND‐V to resolve inflammation at late stages of pneumonia. While in our study, we found CD55^+^ neutrophils accumulate and show activated NETosis at the initial stage of bone aging. These results suggest that the kinetics of CD55 expression on neutrophils orchestrate the dynamic shift between their pro‐ and anti‐inflammatory functions. At the onset of inflammation, CD55 was upregulated on neutrophils to exert pro‐inflammatory functions including NETosis; while during later stages of inflammation, neutrophils age and secrete CD55^+^ LAND‐Vs to resolve inflammation. Therefore, our studies highlight the dynamic functions of neutrophils across different maturation stages and inflammatory contexts.

Furthermore, as NETosis is generally triggered by external stimuli rather than occurring spontaneously [43], we identified the accumulation of ROS in the BM as a potent trigger of NETosis, with senescent BMSCs serving as a source of ROS, consistent with previous studies identifying ROS as a crucial component of the senescence‐associated secretory phenotype (SASP) [44, 45]. Mechanistically, we found ROS integrated with CD55‐primed HIF1α‐PADI4 pathway to activate NETosis, indicating differentiation‐related CD55 converged with environmental oxidative cues to regulate neutrophil function. ROS has been found closely related to HIF1α‐activation. Mitochondrial ROS was found to oxidize PHDs to stabilize HIF1α [46], and hypoxia‐induced HIF1α‐activation can activate NOX to produce ROS, forming positive feedback of ROS generation [47]. Meanwhile, our study verified HIF1α directly upregulated *Padi4* gene expression. Transcription factor HIF1 is composed of α and *β* subunits, and HIF signaling primarily depends on the α subunit [48]. After translocation to the nucleus, it binds to the promoters of target genes containing Hypoxia Response Elements (HREs), initiating gene transcription. Similar with our results, recent studies indicated hypoxia conditions induced direct combination of HIF1 to HREs of PADI4 promoter in breast cancer cells to promote PADI4 expression, and PADI4 protein were also recruited to HREs of other hypoxia‐related genes to stabilize their combination with HIF1α [49]. These results indicated the extensive interactions of HIF1α and PADI4 across different biological processes.

Interestingly, we found NETosis of neutrophils was exclusively activated at the initial stage of aging but inactivated at later stage in both C57BL6/J and SAMP6 mice. Previous research has shown that systemic inflammation activate a pro‐inflammatory function of neutrophils in young individuals. However, neutrophils of aging individuals become immuno‐suppressive as a result of immune exhaustion with impaired NETosis, rendering them less effective at combating pathogens [1, 50]. These biphasic states are consistent with our findings, suggesting during the early stage of bone aging, the inflammatory microenvironment in BM significantly activate neutrophils; while as aging progresses, BM neutrophils become immunosuppressive with inactivated NETosis. Notably, the mechanism underlying late‐stage neutrophil exhaustion still requires further study.

In future, we will further extend our study across a broader range of mouse models and incorporate human samples to validate our findings. First, we will further clarify the “early aging” window in both genders across different ages. Second, we will utilize conditioned knockout mice, including *Padi4*‐knockout mice and conditional *Cd55*‐knockout in neutrophils to validate the phenotype of CD55^+^ neutrophil NETosis and its contribution to bone loss. Third, we will apply human bone marrow samples to verify “early aging” window and confirm the activated NETosis phenotype of CD55^+^ BM neutrophils. In parallel, we will explore the translational potential of our findings by assessing possible clinical readouts of circulating NETs biomarkers, such as MPO‐DNA complexes and citH3, with the ultimate goal of facilitating the early diagnosis of bone aging.

## 4 Conclusions

**FIGURE 8:**
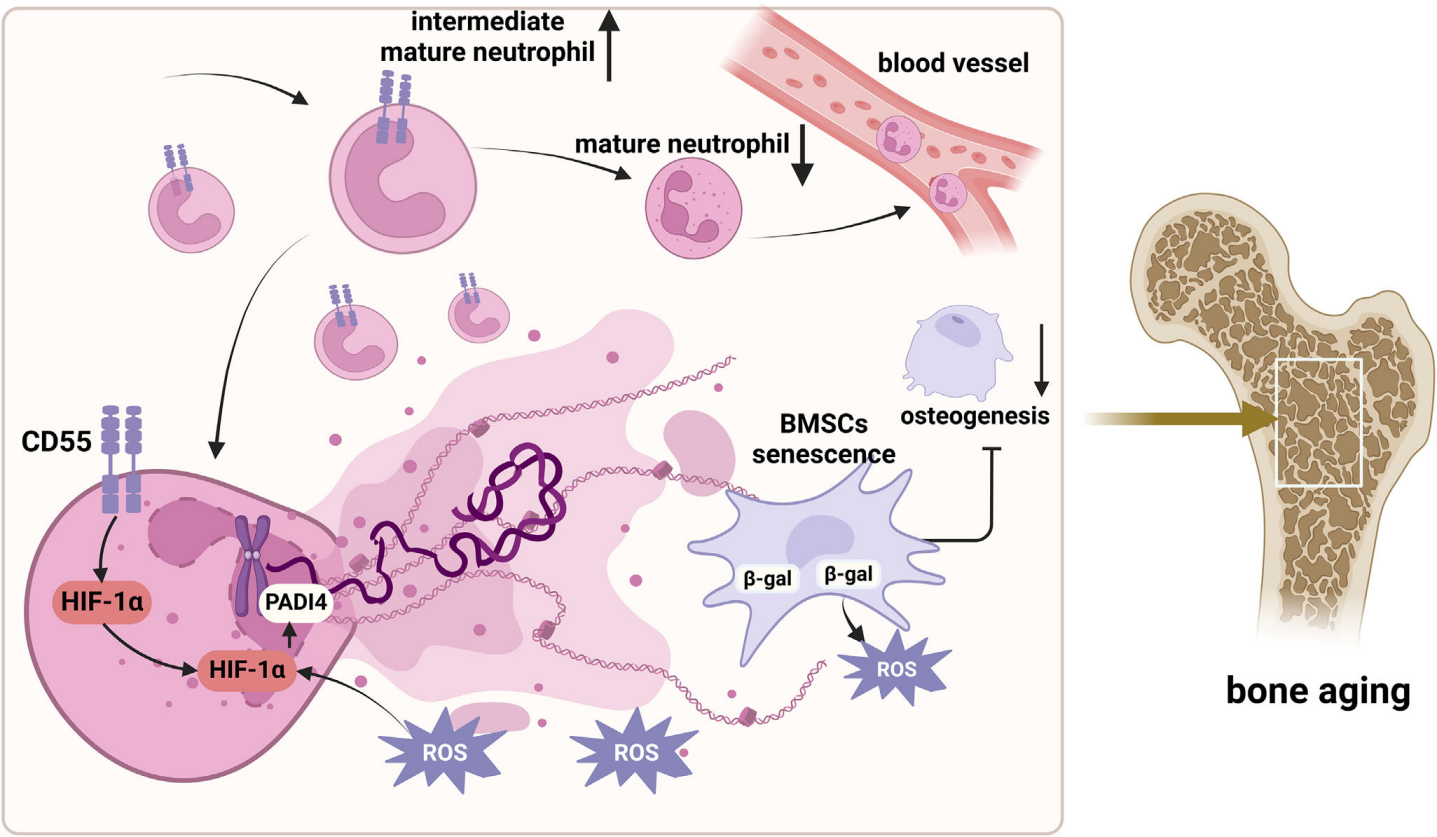
Mechanism scheme: Model illustrating how BM CD55^+^ intermediate mature neutrophils release NETs to induce BMSC senescence and promote bone aging. In 3‐mon male SAMP6 bone marrow, the number of intermediate mature neutrophils increases, while mature neutrophils decrease. Among the intermediate mature neutrophils, CD55^+^ neutrophils are significantly enriched, and CD55 primes NETosis through activation of the HIF1α–PADI4 pathway. ROS accumulating in the BM triggers NETosis through HIF1α–PADI4 pathway. NETs released in BM induce BMSC senescence and inhibit osteogenesis, contributing to bone aging. In turn, senescent BMSCs release more ROS, reinforcing the vicious cycle of inflammaging in the bone marrow.

In this study, we discover an intermediate mature neutrophil subset CD55^+^ neutrophils that accumulate in the BM showing activated NETosis via HIF1ɑ‐PADI4 pathway. NETs further induce BMSCs senescence and osteogenesis inhibition, leading to initiation of bone aging of SAMP6. Meanwhile, senescent BMSCs produce ROS to further activate NETosis through integration with HIF1ɑ‐PADI4 pathway, forming a vicious cycle of inflammaging (Figure 8). These findings provide new insights into the interplay between immune cells and senescent cells in the development of age‐related diseases, and highlight NETosis of CD55^+^ neutrophils as a promising therapeutic target for bone aging treatment.

## 5 Experimental Section

### 5.1 Animals Models

All mice used in this study were appropriately monitored and experimental procedures were conducted in line with guidelines of Animal Use and Care Committee of Peking University with the Animal Welfare Assurance number LA2024060. Mice were housed with a 12/12 light cycle at 72 °F (±2 degrees) and 30% to 70% humidity with ad libitum access to food and water. 8‐week, 3‐mon and 6‐mon male SAMR1 and SAMP6, and 8‐week, 9‐mon and 14‐mon male C57BL6/J mice were purchased from Peking University Health center animal center (Beijing, China). 14‐mon male C57BL6/J mice were bred to 18 months old and then euthanized to harvest their femurs.

For NETs clearance, 8‐week‐old male SAMP6 were injected i.p. with vehicle (DMSO) and/or PADI4 inhibitor Cl‐amidine (20 mg/kg/day) every 3 days for one month and sacrificed at 12‐week‐old. For ROS clearance, 8‐week male SAMP6 were injected with PBS and/or N‐acetylcysteine ip (32 mg/kg) every 2 days for one month and sacrificed at 12 weeks old.

### 5.2 Single‐Cell RNA Sequencing

BM cells of 12‐week male SAMP6 and SAMR1 were respectively flushed from the femur and tibia bones with 3 mL HBSS+2 mM EDTA and filtered through a 70 µm cell strainer. Cells were centrifuged for 5 min at 500 g. To remove red blood cells, 2 mL GEXSCOPE red blood cell lysis buffer (Singleron Biotechnologies, China) was added at 25°C for 10 min. The sample was stained with trypan blue (Sigma) and microscopically evaluated for cell viability and counting, and samples with viabilities >80% were used for sequencing. Single‐cell suspensions with 1×10^5^ cells/mL in concentration in PBS were prepared. Single‐cell suspensions were then loaded onto microfluidic devices and scRNA‐seq libraries were constructed according to Singleron GEXSCOPE protocol by GEXSCOPE Single‐Cell RNA Library Kit (Singleron Biotechnologies) [51]. Individual libraries were diluted to 4 ng/µL and pooled for sequencing. Pools were sequenced on Illumina novaseq6000 with 150 bp paired end reads.

### 5.3 scRNA‐seq Data Processing and Analysis

Raw reads were processed with fastQC and fastp to remove low quality reads. Poly‐A tails and adaptor sequences were removed by cutadapt. After quality control, reads were mapped to the reference genome GRCh38 using STAR. Gene counts and UMI counts were acquired by featureCounts software. Expression matrix files for subsequent analyses were generated based on gene counts and UMI counts.

Seurat was used for quality control, dimensionality reduction, and clustering. Cells with fewer than 500 or more than 6000 detected genes, more than 100000 UMIs, or with more than 10% mitochondrial gene content were excluded. In addition, cells with erythrocyte‐associated genes (*Hba1, Hba2, Hbb, Hbd, Hbe1, Hbg1, Hbg2, Hbm, Hbq1, Hbz*) accounting for more than 3% of total UMIs were removed. Doublets were excluded using *DoubletFinder* (about 5%). After filtering, 13012 SAMR1 cells and 14437 SAMP6 cells were retained for downstream analysis, with 2500–3000 detected genes and 15000–20000 UMIs per cell. Gene expression was normalized with *LogNormalize* and scaled with *ScaleData*. The top 2000 highly variable genes were identified with *FindVariableFeatures* for principal component analysis (PCA). *Harmony* (R package) was applied to correct for batch effects, and Harmony embeddings were used for clustering. Uniform Manifold Approximation and Projection (UMAP) was performed for dimensionality reduction. Differentially expressed genes (DEGs) between different samples or consecutive clusters were identified with function FindMarkers. The cell type identity of each cluster was determined with the expression of canonical markers found in the DEGs combined with knowledge from literature. Heatmaps/dot plots/violin plots displaying the expression of markers used to identify each cell type were generated by Seurat DoHeatmap/DotPlot/Vlnplot. For cellular proportion analysis, the scDC package was used to infer the cell‐type proportion. R package “Slingshot” was used for pseudo‐time analysis, and it performed trajectory inference based on the UMAP dimensionality reduction results as coordinate system for cell distribution [52].

### 5.4 Isolating Bone Marrow Neutrophils

Density‐gradient centrifugation was used to isolate bone marrow neutrophils from SAMP6, SAMR1 and C57BL6/J mice femurs and tibias by “Mouse bone marrow neutrophil isolation solution kit” (Solarbio, China). Briefly, femora and tibias of mice were isolated, and bone marrow was flushed out by RPMI 1640 medium. Then, reagent A, reagent C and bone marrow solution were sequentially added (ratio of 2:1:1.5) to the centrifuge tube to form a gradient interface (880 g, 28 min) according to manufacturers. After centrifugation, granulocytes were gently pipetted between reagent C and reagent A, washed with PBS and then centrifuged. After red blood cells lysed by hemolysate, cells were resuspended with 0.5% BSA for later use. To further isolate neutrophils from granulocytes, 1×10^6^ BM neutrophils were incubated with 1 µL anti‐CD11b‐FITC, and 2 µL anti‐Ly6G‐PE Abs at 4°C for 30 min. After being washed twice with PBS and resuspended in FACS buffer at a concentration of 5×10^6^ cells/mL, Ly6g^+^CD11b^+^ neutrophils were sorted by FACS with BD FACS Aria II sorting equipment (BD biosciences) for further purposes.

### 5.5 Flow Cytometry and Flow Sorting of CD55^+^Ly6g^+^CD11b^+^ Neutrophils

As indicated above, BM cells were flushed out of femur dissected from SAMP6 and SAMR1, and granulocytes were isolated through density‐gradient centrifugation. To further detect CD55 expression and sort CD55^+^Ly6g^+^CD11b^+^ and CD55^−^Ly6g^+^CD11b^+^ neutrophils, 1×10^6^ cells were blocked with 1% BSA and then stained with 1 µL anti‐CD11b‐FITC, 2 µL anti‐Ly6G‐PE and 2 µL anti‐CD55‐APC Abs in 100 µL FACS buffer at 4°C for 30 min. To detect CXCR2 and CXCR4 expression of BM neutrophils, 1×10^6^ cells were blocked with 1% BSA and then stained with 1 µL anti‐CD11b‐FITC, 2 µL anti‐Ly6G‐PE, 2 µL anti‐CD55‐APC, 2 µL anti‐CXCR4‐PERCP or 2 µL anti‐CXCR2‐PERCP Abs in 100 µL FACS buffer at 4°C for 30 min. After two washes with PBS and then centrifugation, these cells were ready for FACS analysis (NovoExpress, Agilent, United States) or flow sorting (Aria II, BD Bioscience, United States).

### 5.6 In Vitro Stimulation of NETs Release of BM Neutrophils

BM neutrophils (1×10^6^ cells/mL) were incubated in poly‐L‐lysine coated on coverslips on 12‐well tissue culture plates, and 100 nM PMA or 100 µM H_2_O_2_ was administrated for 4 h to stimulate NETs release of neutrophils. After stimulation, cells were used for immunofluorescence. For NETs isolation, micrococcal nuclease (0.3 U/mL) were added to neutrophils for NETs detachment from cells for 30 min at 37°C. Then cells were centrifuged at 200 g for 5 min. The NETs‐containing supernatant was stored at −80°C or directly used.

### 5.7 SAMR1 Transferred With Neutrophils

For neutrophils transferring, 8‐week male SAMR1 were injected via tail vein with one million CD55^+^ neutrophils or BM neutrophils of 3‐mon male SAMP6 every 5 days for a total of 5 injections and were sacrificed at 12‐week‐old. Specifically, 3‐mon male SAMP6 mice were sacrificed for isolation of CD55^+^Ly6G^+^CD11b^+^ and Ly6G^+^CD11b^+^ neutrophils as the methods indicated above. BM neutrophil cells were first enriched through density‐gradient centrifugation, and then CD55^+^Ly6G^+^CD11b^+^ neutrophils were sorted by BD FACS Aria II (BD biosciences, United States). The purity of CD55^+^ neutrophils and Ly6G^+^CD11b^+^ neutrophils in post‐sort fractions was over 90%, which was confirmed using a BD FACS LSRII cytometer. After cell sorting, 8‐week‐old male SAMR1 were immediately injected with one million CD55^+^ neutrophils suspended in 200 µL RPMI 1640 medium with 1% penicillin‐streptomycin per mice via tail vein, one time every 5 days for a total of 6 injections. Positive control group of 8‐week male SAMR1 were injected with the same amount of Ly6G^+^CD11b^+^ neutrophils from SAMP6, blank group was injected with 200 µL RPMI 1640 medium with 1% penicillin‐streptomycin. All groups were sacrificed after 4 weeks at 3‐mon old.

### 5.8 Co‐Culture of NETs and BMSCs

As described above, NETs released by BM neutrophils were isolated and collected. NETs released by 1×10^6 ^BM neutrophils of 3‐mon male SAMP6 or 9‐mon male C57BL6/J were collected, and the supernatant was in 500 µL RPMI 1640 medium. Nanodrop spectrophotometer (Thermo Fisher Scientific, USA) quantified DNA contained in collected supernatant and were further adjusted to 1500 ng/µL. For stimulation of BMSCs, 250 µL NETs supernatant was added to 5×10^5^ P2 BMSCs per 24 h in 2 mL complete medium for consecutive 2 days. For DNA disruption of NETs, 10 U/mL DNase I was added to NETs supernatant and incubated for 30 min at 37°C. Then DNA‐disruption effect was confirmed by Nanodrop. Then 250 µL processed NETs supernatant was added to 5×10^5^ P2 BMSCs per 24 h for consecutive 2 days.

### 5.9 Dual Luciferase Reporter Assay

First, we transfected HL‐60 cells with the pGL3‐Basic, pGL3‐PADI4‐WT, and pGL3‐PADI4‐MUT plasmids (constructed by Qsingke Biotechnology, China), HIF1α‐OE and EV plasmids (Sinobiological, China), and the Renilla luciferase (pRL‐SV40) reporter gene plasmid (Beyotime, China) in 6‐well plates. We ensured the HL‐60 cells concentration at least 1×10^7^/mL in wells. Then we mixed 125 µL of Opti‐MEM Medium without antibiotics, 2.5 µg of plasmid DNA (or 0.5 µg of pRL‐SV40) and 4 µL of Lipo8000 transfection reagent in EP tube, and added 125 µL of the mixture into the HL‐60 cells and transfected for 48 h. After transfection, we aspirated cells from the 6‐well plates, centrifugated, and then performed the dual luciferase reporter assay using Dual Luciferase Reporter Gene Assay Kit (Yeasen, China) according to manufacturer's instruction. Briefly, cell pellets were lysed by lysis buffer, and 20 µL of the lysate of was added to a 96‐well opaque plate. Then the firefly luciferase reaction working solution were added and incubated at room temperature before performing analysis using multimode plate reader (Revvity, USA). Then Renilla luciferase reaction solution was added and analyzed. Then data was further processed by Graphpad Prism 10.

### 5.10 ROS Staining of Femur BM

To detect ROS level within mice femur BM, we used the CellROX Green fluorogenic probe (Thermo Fisher Scientific, USA) which has been verified to withstand PFA (paraformaldehyde) fixation. First, we isolated mice femurs and removed a small portion of metaphysis at one end. Then we used a 1 mL syringe to aspirate 100 µL of CellROX Green working solution and carefully injected it into the bone marrow cavity through the needle inserted into the femur, ensuring no leakage occurred. The femurs were then immersed in CellROX Green working solution overnight, 4°C and protected from light. Subsequently, the femurs were fixed in 4% PFA for 48 h, followed by decalcification and section. The remaining procedures were consistent with standard tissue immunofluorescence staining protocols indicated in Supplementary information.

### 5.11 Establishment of a CM–Neutrophil Co‐Culture Model

To collect the conditional medium (CM) of BMSC, BMSCs were isolated from the femurs of 3‐mon male SAMP6 mice and cultured to passage 2 (P2). The culture medium from P2 BMSCs in a 10‐ cm dish (approximately 10 mL) was collected and centrifuged at 2000 rpm for 5 min, and the supernatant was then collected as CM. To establish the co‐culture model, BM neutrophils were isolated and sorted by flow cytometry from 3‐mon male SAMP6 mice. Neutrophils were seeded at a density of 5×10^5^cells/mL into 24‐well plates containing coverslips. The experiment included three groups: (1) H_2_O_2_ group: neutrophils were cultured in 1 mL of 10% RPMI‐1640 complete medium supplemented with 100 µM H_2_O_2_; (2) CM group: neutrophils were cultured in 1 mL of BMSC‐CM; (3) CM + NAC group: neutrophils were cultured in 1 mL of BMSC‐CM supplemented with 100 µM N‐acetylcysteine (NAC). After 8 h of treatment, cells were harvested for immunofluorescence staining to detect NETs release.

Details in reagents and methods including Micro‐CT and bone histomorphometric analysis, visualization of NETs by fluorescence microscopy, BMSCs isolation and culture, senescence‐associated *β*‐gal staining, histochemistry, immunohistochemistry and immunofluorescence staining, osteogenic differentiation assay, siRNA transfection, neutrophil‐induction and flow cytometry of HL‐60 cells, qRT‐PCR analysis, western blotting (WB) were included in Supplementary File. Primer sequences are listed in Table S2.

### 5.12 Statistical Analysis

All results are expressed as the mean ± SD. Variances were similar between groups for most parameters assessed. Comparisons between two groups were analyzed using Student's two‐tailed unpaired *t* test and those among 3 or more groups using one‐way analysis of variance (ANOVA) followed by Tukey's post hoc multiple comparisons. p values<0.05 were considered statistically significant. Statistical data in this study were analyzed, and dot plots were generated using GraphPad Prism 10.

## Author Contributions

Y.G. contributed to conception and design, data acquisition, analysis, and interpretation, drafted and critically revised the manuscript. S.C., X.W., and Y.W. contributed to analysis, and interpretation. B.W. contributed to data analysis and interpretation of scRNA‐seq data. Y.W. and Y.G. contributed to conception and design, data analysis, drafted and critically revised the manuscript.

## Funding

This work was supported by National Natural Science Foundation of China (grant Nos. 81970979, 81970920, 82101043) and Natural Science Foundation of Beijing Municipality, China (grant Nos. 7232217, 7242282, 7232218).

## Conflicts of Interest

The authors declare no conflicts of interest.

## Supporting information




**Supporting File**: advs73712‐sup‐0001‐SuppMat.docx.

## Data Availability

The data that support the findings of this study are available from the corresponding author upon reasonable request.
