# Supplementary material for: ROS Activated NETosis of Bone Marrow CD55+ Intermediate Mature Neutrophils Through HIF1α‐PADI4 Pathway to Initiate Bone Aging
Source: Adv Sci (Weinh). 2026 Jan 8;13(16):e00046. doi: 10.1002/advs.202500046 (PMC13042873; doi:10.1002/advs.202500046)
Supplement: Supplementary file 1 — Supporting File: advs73712‐sup‐0001‐SuppMat.docx. [file ADVS-13-e00046-s001.docx]

Supporting Information

**ROS Activated NETosis of Bone Marrow CD55^+^ Intermediate Mature Neutrophils through HIF1α-PADI4 Pathway to Initiate Bone Aging**

Yutong Guo^a^, Shengjie Cui^b^, Xi Wen^a^, Yidi Wang^a^, Ben Wu^c*^, Yixiang Wang^d*^, Yan Gu^a*^

Correspondence:

guyan96@126.com; kqwangyx@bjmu.edu.cn; wuben@ruc.edu.cn

**This file includes:**

1. Supplementary Figures and Legends

2. Table S1, S2, S3.

3. Supplementary Methods.

Figure S1. Initiation of bone aging of SAMP6 and C57BL6/J mice.

**A.** μCT analysis indicated femur bone mass of 8-week male SAMP6 was comparable with 8-week SAMR1. **B.** Quantitative μCT analysis showed BMD, BV/TV, Tb.th of distal end of femurs of 8-week SAMP6 of no significant difference with 8-week SAMR1. n=5 mice per group. **C.** Immunofluorescence indicated up-regulated γH2A.X in 3-mon SAMP6 BM than SAMR1. Scale bar, 20 μm. n = 5 biologically independent samples. **D.** Semi-quantitative analysis of WB results of Figure 1E, performed using ImageJ software to measure the grayscale area (n = 3). **E.** β-gal^+^ cells of Figure 1F were analyzed by Image J in randomly chosen microscope views(n=5). **F.** mRNA levels of *Runx2*, *Bglap*, *Sp7* were significantly lower in BMSCs isolated from 3-mon SAMP6 than SAMR1 after osteogenic induction for 14 days. **G.** μCT analysis indicated significantly decreased trabecular bone of 9-mon C57BL6/J male mice than 8-week counterpart. **H.** Quantitative μCT analysis of distal end of femurs indicated 9-mon mice with lower BMD, BV/TV, Tb.th than 8-week mice. n=5 mice per group. **I.** H&E analysis indicated fewer trabecular bone from the femur metaphysic in 9-mon mice femur. **J.** Immunofluorescence indicated elevated γH2A.X expression in BM of 9-mon C57BL6/J mice than that of 8-week. **K.** β-gal staining showed BMSCs isolated from 9-mon C57BL6/J mice with higher senescence than that of 8-week. Scale bar, 100 μm. **L.** Double-positive citH3^+^Ly6G^+^ cells of Figure 1J were analyzed by ImageJ in randomly chosen microscope views. n = 5 biologically independent samples. NE and MPO expression of Figure 1I was analyzed by Image J. n = 3 biologically independent samples. NETs-released neutrophils of Figure 1K were calculated by Image J. n = 5 biologically independent samples. **M.** Flow sorting strategy of Ly6G^+^CD11b^+^ neutrophils of BM of SAMP6 and SAMR1. Data were represented as mean ± SD. **N.** NETs-released neutrophils of Figure 1L were calculated by Image J. n = 3 biologically independent samples. Data were analyzed by two-tailed Student’s *t* test or one-way ANOVA with Tukey’s post hoc test. ns, not significant, **P < 0.01, ***P < 0.001, ****P < 0.0001.

Figure S2. BM neutrophil NETosis of 9-mon male C57BL6/J mice and 3-mon SAMP6 was activated.

**A.** Immunohistochemical staining indicated higher expression of MPO and NE in 9-mon mice BM than 8-week mice, with scale bar of 100 μm. **B.** Immunofluorescence staining indicated higher expression of citH3 in 9-mon mice BM. Scale bar of 50 μm. **C.** Flow sorting of Ly6G^+^CD11b^+^ BM neutrophils from C57BL6/J mice. **D.** Semi-quantitative analysis of WB results of Figure 1N, performed using ImageJ software to measure the grayscale area (n = 3). **E.** β-gal^+^ cells of Figure 1O were analyzed by Image J in randomly chosen microscope views(n=5). **F.** ALP and ARS staining indicated NETs-stimulation inhibited osteogenesis of BMSCs, while DNase I recued their effects. **G.** mRNA levels of *Runx2*, *Bglap*, *Sp7* were significantly inhibited by NETs-stimulation to BMSCs, while DNase I recued their effects. **H.** β-gal staining indicated NETs of BM neutrophils of 9-mon C57BL6/J induced BMSCs senescence. Scale bar, 100μm. **I.** Fluorescence indicated NETs of BM neutrophils of 9-mon C57BL6/J mice elevated ROS expression of BMSCs. **J.** ALP and ARS staining indicated NETs of BM neutrophils of 9-mon C57BL6/J mice inhibited osteogenesis of BMSCs. **K.** PCR indicated inhibition of STING through siRNA transfection of STING. **L.** WB analysis indicated NETs activated cGAS, STING, p21, p16 expression of BMSCs *in vitro*, and transfection of STING siRNA abrogated these effects. **M.** Semi-quantitative analysis of WB results of Figure S2L, performed using ImageJ software to measure the grayscale area (n = 3). **N.** β-gal staining showed senescence-inducing effects of NETs was abrogated by silencing STING through siRNA transfection (n = 5 biologically). Data were analyzed by two-tailed Student’s *t* test or one-way ANOVA with Tukey’s post hoc test. **P < 0.01, ***P < 0.001, ****P < 0.0001.

Figure S3. Early NETs-clearance ameliorates bone aging of 3-mon SAMP6.

**A.** Double-positive citH3^+^Ly6G^+^ cells of Figure 2G were analyzed by ImageJ in randomly chosen microscope views. n = 5 biologically independent samples. n = 5 biologically independent samples. **B.** NETs-released neutrophils of Figure 2H were calculated by Image J. n = 5 biologically independent samples. **C.** γH2A.X^+^ cells of Figure 2I were analyzed by Image J in randomly chosen microscope views(n=5). **D.** β-gal^+^ cells of Figure 2J were analyzed by Image J in randomly chosen microscope views(n=5). **E.** Semi-quantitative analysis of WB results of Figure 2K, performed using ImageJ software to measure the grayscale area (n = 3). **F.** After osteogenic induction for 14 days *in vitro*, BMSCs isolated from Cl-amidine-recipient mice showed higher mRNA levels of *Runx2*, *Bglap*, *Sp7* of BMSCs than SAMP6 mice. Data were analyzed by two-tailed Student’s t test. **P < 0.01, ***P < 0.001, ***P < 0.0001.

Figure S4. Early NETs-clearance ameliorates bone aging of 3-mon SAMP6.

A. The violin plots showed the distribution of feature_RNA, count_RNA and mitochondrial RNA proportions of SAMR1 and SAMP6 samples. The results show that most cells have 500-6000 detected genes (nFeature_RNA), with very few outliers below 200. The majority of cells have fewer than 40,000 transcripts (nCount_RNA), and mitochondrial gene content is below 10% in most cells. These metrics indicate high sample quality with minimal contamination by low-quality or apoptotic cells. **B.** FeatureScatter plot revealed the relationship between Feature_RNA and count_RNA. Gene and transcript counts are comparable between SAMP6 and SAMR1 samples.nFeature_RNA and nCount_RNA show a strong positive correlation, with Pearson coefficients >0.9 in both groups. **C.** The variable feature plot showed the expression levels of all genes in all cells, and the 2000 genes with the most variable value were selected for subsequent analysis, and the top 10 genes were marked.

Figure S5. BM cell clusters-specific gene expression.

**A.** Feature plot showed the expression of marker genes in different BM cell populations. **B.** Dotplot indicated expression of marker gene we chose for BM cell-clustering. **C.** Heatmap indicated top 5 DEGs of BM cell clusters.

Figure S6. Gene expression and maturation stage of CD55^+^ neutrophils.

**A.** UMAP indicated clustering of BM neutrophils. **B.** Projection of differentiation trajectory, neutrophil subsets with cells of SAMP6 and SAMR1. The asterisk represents C10, primarily composed of cells from SAMP6 mice. **C.** Feature plot indicated CXCR4 and CXCR2 expression in neutrophil subsets, with CXCR4 mainly in the middle stage of differentiation while CXCR2 mainly at late stage. **D.** Violin plot indicated neutrophil differentiation-related gene Ly6G, Kit and CEBP/e expression in neutrophils subsets in the order of maturation, indicating C10 in middle of maturation. **E.** Heatmap of top 10 DEGs in 9 neutrophil subsets. Yellow indicated highly expressed genes. **F.** Dot plots indicate that CD55 expression is upregulated in C10, compared with C7 and other subclusters. **G**. KEGG analysis indicated NETs-formation, ROS-related pathway and HIF1-related pathway significantly activated in neutrophil subset C10. **H.** GO analysis of CD55-related pathway in C10 indicated it was mainly involved in cell cycle and differentiation related pathways.

Figure S7. CD55^+^Ly6G^+^CD11b^+^ cells are identified as intermediate mature neutrophils.

**A.** Statistical analysis results of flow cytometry in Figure 4B. 3 biological replicates. **B**. Flow cytometry analysis indicated BM neutrophils (Ly6G^+^CD11b^+^) of 9-mon male C57BL6/J mice showed higher expression of CD55 than 8-week mice. Also, CXCR2 showed low expression while CXCR4 of high expression in CD55^+^ neutrophils of both 8-week and 9-mon male C57BL6/J mice. **C.** Fluorescence staining indicated 9-mon male C57BL6/J mice contained more CD55^+^Ly6G^+^ cells in BM than 8-week mice. Scale bar, 50μm. **D.** Gating strategy of CXCR2 and CXCR4 expression analysis of CD55^+^Ly6G^+^CD11b^+^ cells of 3-mon SAMR1. **E.** Statistical analysis results of Figure 4C. 3 biological replicates. **F.** Statistical analysis results of Figure 4D. 3 biological replicates. **G.** During HL-60 differentiation towards neutrophils from day 0 to day 5 and post-maturation from day 5 - day 7, CD11b^+^ neutrophils gradually increased, and CD55 expression in CD11b^+^ neutrophils peaked at day 4. Data were analyzed by two-tailed Student’s *t* test. ns, not significant, *P<0.05, **P < 0.01, ***P < 0.001, ***P < 0.0001.

Figure S8. CD55 activated neutrophil NETosis via CD55-HIF1α-PADI4 pathway.

**A.** Immunofluorescence indicated CD55 significantly co-localized with NETs-released neutrophils. Scale bar of 50 μm. **B.** Semi-quantitative analysis of WB results of Figure 5B, performed using ImageJ software to measure the grayscale area (n = 3). **C.** CD55 and HIF1α mRNA was significantly upregulated after over-expression plasmid transfection. CD55 and HIF1α mRNA was significantly inhibited after siRNA transfection. **D.** Semi-quantitative analysis of WB results of Figure 5C, performed using ImageJ software to measure the grayscale area (n = 3). **E.** HIF1α-OE plasmid transfection was also confirmed by the GFP expression in HL-60 cells. **F.** Semi-quantitative analysis of WB results of Figure 5D, performed using ImageJ software to measure the grayscale area (n = 3). Data were analyzed by two-tailed Student’s *t* test or one-way ANOVA with Tukey’s post hoc test. **P < 0.01, ***P < 0.001, ***P < 0.0001.

Figure S9. Cell transferring of CD55^+^ neutrophils to SAMR1 mice.

A. Double-positive CD55^+^Ly6G^+^ cells of Figure 6B were analyzed by ImageJ in randomly chosen microscope views. n = 5 biologically independent samples. **B.** Number of TRAP^+^ osteoclasts in distal marrow per tissue areas (n = 5) of Figure 6D. **C.** NE and MPO expression of Figure 6E-F was analyzed by Image J. n = 3 biologically independent samples. Double-positive citH3^+^Ly6G^+^ cells of Figure 6G were analyzed by ImageJ in randomly chosen microscope views. n = 5 biologically independent samples. **D.** NETs-released neutrophils of Figure 6H were calculated by Image J. n = 5 biologically independent samples. **E.** β-gal^+^ cells of Figure 6I were analyzed by Image J in randomly chosen microscope views(n=5). **F.** Semi-quantitative analysis of WB results of Figure 6J, performed using ImageJ software to measure the grayscale area (n = 3). Data were analyzed by two-tailed Student’s *t* test. *P < 0.05, **P < 0.01, ****P < 0.0001.

Figure S10. ROS-clearance through NAC in 3-mon SAMP6.

**A.** Flow cytometry indicated increased intracellular ROS in 3-mon SAMP6 BM cells than SAMR1. **B.** Fluorescence staining indicated higher spontaneous ROS expression in the BM of 9-mon male C57BL6/J mice than 8-week mice. **C.** Flow cytometry indicated increased intracellular ROS in 3-mon SAMP6 BMSCs than SAMR1. **D.** BMSCs markers SOX9 and RUNX2 showed less expression but more significant co-localization with spontaneous ROS in the BM of SAMP6 mice than SAMR1. Scale bar, 50 μm. **E.** H_2_O_2_ activated MPO, PADI4 expression of HL-60 while the effects were abrogated by ROS scavenger NAC. **F.** ROS^+^ cells of were analyzed by Image J in Figure 7H randomly chosen microscope views(n=5). **G.** NE and MPO expression of Figure 7I was analyzed by Image J. n = 3 biologically independent samples. Double-positive citH3^+^Ly6G^+^ cells of Figure 7J were analyzed by ImageJ in randomly chosen microscope views. n = 5 biologically independent samples. **H.** NETs-released neutrophils of Figure 7K were calculated by Image J. n = 5 biologically independent samples. **I.** Semi-quantitative analysis of WB results of Figure 7L, performed using ImageJ software to measure the grayscale area (n = 3). **J.** μCT analysis of distal femurs, trabecular bone indicated bone mass of SAMP6 mice was rescued after injection of NAC. Quantitative μCT analysis of distal end of femurs (n = 5) indicated NAC injection significantly increased BMD, BV/TV, Tb.th, Tb.N and decreased Tb.Sp of SAMP6 mice. **K.** H&E analysis showed significantly more trabecular bone after NAC injection. Scale bar, 200 μm. Data were analyzed by two-tailed Student’s *t* test. *P < 0.05, **P < 0.01, ***P < 0.001,****P < 0.0001.

**Table S1. Proportion of neutrophil subsets in SAMP6 and SAMR1 BM.**

|  | C0 | C1 | C2 | C7 | C6 | **C10** | C12 | C17 | C20 |
| --- | --- | --- | --- | --- | --- | --- | --- | --- | --- |
| SAMR1  (%) | 17.85 | 14.26 | 15.69 | 21.26 | 10.20 | **1.48** | 8.68 | 6.18 | 4.40 |
| SAMP6  (%) | 26.27 | 21.98 | 13.06 | 0.92 | 11.01 | **14.50** | 5.77 | 3.28 | 3.20 |
| SAMP6/SAMR1(fold) | 1.47 | 1.54 | 0.83 | 0.04 | 1.08 | **9.80** | 0.66 | 0.53 | 0.73 |

According to scRNA-seq data, SAMP6 and SAMR1 BM contained different proportions of neutrophils, with C10 significantly infiltrating in SAMP6 BM with 9.76-fold than that of SAMR1.

**Table S2. Nucleotide sequences of primers used for mice and human cells quantitative RT-PCR detection.**

| **Primers** | **Sequence (5' to 3')** |
| --- | --- |
| *Runx2*(mouse) | F: ATGTCCGCCACCACTCACTAC |
|  | R: GGAACTGATAGGATGCTGACGAAG |
| *Bglap*(mouse) | F: AGCAGGAGGGCAATAAGGTAGTG |
|  | R: CTCGTCACAAGCAGGGTTAAGC |
| *Sp7*(mouse) | F: TCACCTGCCTGCTCTGTTCC |
|  | R: CGGCTGATTGGCTTCTTCTTCC |
| *Sting* (mouse) | F: CCAAGAACCCACAGACGGAAAC |
|  | R: GAGGAGGTGCCACTGAGGTC |
| CD55(human) | F: TGCTGCTGCTGGTGCTGTTG |
|  | R: GCTGGCTGGGCATTAGGTACATC |
| HIF1α (human) | F：CTAACTAGCCGAGGAAGAACTATGAAC |
|  | R：CACTGAGGTTGGTTACTGTTGGTATC |
| GAPDH (human) | F: ACACCCACTCCTCCACCTTTG |
|  | R: TCCACCACCCTGTTGCTGTAG |
| Gapdh (mouse) | F: AGGTCGGTGTGAACGGATTTG |
|  | R: GGGGTCGTTGATGGCAACA |

**Table S3. Summary of markers and characteristics of CD55^+^ BM neutrophils.**

| CD55^+^ BM neutrophils | Markers |
| --- | --- |
| Flow sorting | CD55⁺Ly6G⁺CD11b⁺ |
| Maturation | CXCR4^hi^CXCR2^int^ |
| Transcriptional hallmarks | *Mpo; Elane; Padi4; Hif1a* |
| Published nomenclature ^[6]^ | Intermediate mature neutrophils,  stage between G3-G4 |

**Table S4. Nucleotide sequences of siRNA used for mice and human cells.**

| siRNA | **Sequence (5' to 3')** |
| --- | --- |
| *si-STING* (mouse) | F: GGUCAUACUACAUUGGGUATT |
|  | R: UACCCAAUGUAGUAUGACCTT |
| *si-CD55* (human) | F: UGAAGAAAGCUUUGUGAAATT |
|  | R: UUUCACAAAGCUUUCUUCATT |
| *si-HIF-1ɑ* (human) | F: CAGAAAUGGCCUUGUGAAATT |
|  | R: UUUCACAAGGCCAUUUCUGTT |

**Supplementary** **Methods**

**Reagents**

The following Abs were purchased from Abcam Inc. (England): anti-CD11b(ab133357), anti-Histone H3 (citrulline R2 + R8 + R17) antibody (ab281584), anti-PADI4(ab96758), anti-CDKN2A/p16INK4a antibody (EPR20418) (ab211542), anti-p21 antibody (ab109520, ab188224), anti-HIF1ɑ(ab51608). STING (D2P2F) Rabbit mAb (13647), cGAS Rabbit mAb (31659), cGAS Rabbit mAb (79978) and Ly-6G Rabbit mAb (87048) were from Cell Signaling Technology (United States). Neutrophil elastase antibody (AF0010), PADI4 antibody (DF6685) and histone H3 antibody (AF0863) were from Affinity Biosciences (China). MPO antibody(22225-1-AP), CD55 antibody (26580-1-AP) and cleaved-caspase 3 antibody (25128-1-AP) were purchased from Proteintech (China). PE anti-mouse Ly-6G antibody (127607), FITC anti-mouse/human CD11b antibody (101205), Alexa Fluor® 647 anti-mouse CD55 (131806), APC anti-human CD55 antibody (311311), PerCP/Cyanine5.5 anti-mouse CD184 (CXCR4) antibody (146509) and PerCP/Cyanine5.5 anti-mouse CD182 (CXCR2) antibody (149307) were purchased from Biolegend (United States). Primary and secondary Abs for flow cytometry were used at 1:100 dilution. Acetylcysteine (N-acetylcysteine, S1623) was purchased from Selleck Chemicals (United States). Clamidine (10599) was purchased from Cayman chemical. PMA (P8139) and DMSO (D8418) were purchased from Sigma-Aldrich (United States). Poly-D-lysine and dihydroethidium (DHE, ST508), and micrococcal nuclease (D7201S) were from Beyotime (China). Hydrogen peroxide (AAT-A11004) was from AAT Bioquest (United States). Goat anti-Rabbit IgG-FITC (SF134) secondary antibody, Goat Anti-Rabbit IgG/RBITC (SR134) secondary antibody, bone marrow neutrophil Isolation Kit (P8550) were purchased from Solarbio (China).

**µCT and bone histomorphometric analysis**

Femurs of SAMP6 and SAMR1 mice were isolated fixed in 4% paraformaldehyde for 24h. µCT scanning with a resolution of 8 µm was performed using high resolution µCT (Skyscan 1276, Bruker MicroCT, Kontich, Belgium), and trabecular bone parameters were assessed in a region 1-4 mm from the edge of the growth plate in the metaphyses of femora. Parameters of the trabecular bone in the metaphysis and cortical bone in the mid-diaphysis were analyzed using NRecon image reconstruction software, version 2.2.0.6 (Bruker MicroCT), CTAn data-analysis software, version 1.20 (Bruker MicroCT), and CTVol 3-dimensional model visualization software, version 2.0 (Bruker MicroCT). The scanner was set at 50 kV, 201 mA and a resolution of 8 µm. For the distal femur, the region of interest (ROI) selected for analysis was 5% of the femoral length from 0.1mm below the growth plate to determine trabecular bone volume per tissue volume (BV/TV), trabecular number (Tb.N), trabecular separation (Tb.Sp), and trabecular thickness (Tb.Th).

**Visualization of NETs by fluorescence microscopy**

Neutrophils seeded on coverslips were fixed with 4% PFA, permeabilized with 0.3% Triton X-100 for 5 min, followed by blocking with 10% BSA for 30 min. Cells were stained with antibodies against MPO/NE/CD55/HIF1ɑ overnight in the dark at 4℃. The next day, Goat anti-Rabbit IgG-FITC or Goat Anti-Rabbit IgG-RBITC or/and Goat Anti-mouse IgG-FITC secondary Ab was applied for incubation for 1 h under room temperature. At last, DAPI was stained for 5 min. After mounting, cells were visualized using a con-focal FV3000 microscope (Olympus, Japan).

**BMSCs isolation and culture**

BMSCs were isolated from bilateral tibiae and femurs of respectively 2 SAMP6 and SAMR1 mice each time. Briefly, BM cells were flushed out with 2 mL α-MEM with 10% FBS, filtered through a 100-μm cell strainer (BD Falcon, United States) and then seeded on TCPs. After 24 h, the medium was replaced to remove non-adherent cells. The medium was then changed every 48 h and then passaged to P2 for later experiment.

**Senescence-associated β-gal staining**

BMSCs seeded onto 24-well plates were fixed and stained according to the instructions of the senescence-associated β-galactosidase detection kit (Cell Signalling Technology, United States). Briefly, cells were washed with PBS and fixed with 1×Fixative Solution for 10 min. Senescence-associated β-galactosidase (SA-β-gal) staining solution was added to stain senescent cells and incubated at 37°C in a dry incubator for overnight. Five fields of view were randomly selected for observation under an optical microscope (Nikon, Japan).

**Cellular ROS staining for flow cytometry and fluorescence**

We used the ROS fluorescence probe DCFH-DA of Reactive Oxygen Species Assay Kit (Beyotime, China) to detect cellular ROS level. First, we diluted DCFH-DA in serum-free ɑ-MEM medium at a ratio of 1:1000 to achieve a final concentration of 10 μM. Then we suspended mice femoral BMSCs or BM cells in the diluted DCFH-DA solution in EP tubes at a concentration of 5×10^6^ cells/mL, and incubated cells at 37 °C in a cell incubator for 20 min. Next, we centrifuged cells at 300 g for 5 minutes, discard the supernatant, and washed the cells three times with PBS to fully remove extra DCFH-DA. Finally, we suspended the cells in 500 PBS, filtered through a cell strainer, and analyze using the NovoExpress flow cytometer using the FITC channel to detect ROS levels. For ROS fluorescence staining of BMSCs seeded on TCPs, we also used the Reactive Oxygen Species Assay Kit. We first removed the culture medium from TCPs and washed cells with PBS, then incubated cells with 10 μM DCFH-DA solution sufficient to cover the cells for 20 min at 37 °C, following three washes with PBS to remove extracellular probe. Finally, intracellular ROS were assessed by fluorescence microscopy (excitation 488 nm/emission 525 nm) (Olympus, Japan).

**Histochemistry, immunohistochemistry and immunofluorescence staining**

Femora of mice were harvested after euthanasia, fixed in 4% PFA (paraformaldehyde) for 24 h, decalcified in 10% EDTA for 28 days in room temperature, and embedded in paraffin. Tissue sections were prepared longitudinally with a paraffin microtome (Leica, Germany). Four-μm-thick oriented bone sections were stained with hematoxylin-eosin (HE, Solarbio) and TRAP (Solarbio, China) to indicate trabecular bone and number of osteoclasts in BM. For immunohistochemical staining, after xylene deparaffinization and ethanol rehydration, bone sections were processed for antigen retrieval by digestion with 0.125% trypsin and 20 μg/mL proteinase K solution incubated at 37 °C for a 30-min period. Endogenous peroxidase activity was blocked with 3% H_2_O_2_ for a 30-min period at room temperature, washed with PBS and blocked with 5% BSA for 1 h. Then sections were incubated with primary antibody against MPO, NE overnight at 4℃. Subsequently, an HRP-streptavidin detection system was used to detect the immunoactivity, followed by counterstaining with hematoxylin (Solarbio, China).

For immunofluorescence, heat-induced antigen retrieval with sodium citrate buffer (10 mM sodium citrate, 0.05% Tween 20, pH 6.0) was performed before bone sections were blocked with 5% BSA for 1 h at room temperature, then incubated overnight at 4 °C with primary antibodies diluted in PBS with 1% BSA. Subsequently, sections were incubated with secondary fluorescent conjugated antibodies at room temperature for 1 h in the dark. Samples were imaged by the FV3000 confocal microscope (Olympus, Japan).

**Osteogenic differentiation assay**

For osteoblastic differentiation, P2 BMSCs were cultured in 12-well plates with α-MEM containing 10% fetal bovine serum, ascorbic acid (final concentration of 50 μg/mL), β-glycerophosphate (final concentration of 5 mM), and dexamethasone (final concentration of 100 nM). Cells were stained with ALP staining (Beyotime) after osteogenic induction of 7 days according to the instructions of the ALP staining kit (Beyotime). Briefly, cells were fixed with 4% paraformaldehyde and washed with PBS. Then, ALP staining solution was added and stained for 30min. Cells were stained with 1% Alizarin Red (Solarbio) after osteogenic induction for 14 days. Briefly, cells were fixed with 4% PFA, and 1% ARS staining solution was added and co-incubated with cells for 10 min (Solarbio). After washing with PBS, the formation of mineralized nodules in the cells of each group was observed under an optical microscope and photographed (Nikon, Japan).

**siRNA and plasmid transfection**

To knockdown STING in mBMSCs and CD55 in HL-60 cells, siRNA transfection was performed according to the manufacturer’s instructions. The siRNA oligonucleotides or siRNA control were transfected on 6/12/24-well plates in the presence of 10% fetal bovine serum using Lipofectamine™ 3000 (L3000015, Thermo Fisher Scientific, United States). All siRNA products were purchased from the LncBio Company (China). The targeting sequences for mouse STING knockdown were included in Table S4. Efficiency of knockdown 48 h (n = 3) after transfection was evaluated by qRT-PCR and WB.

Plasmid transfection was performed for CD55 and HIF1ɑ over-expression in HL-60 cells according to the manufacturer’s instructions. All plasmid products were purchased from Sinobiological Company (China). The vector pCMV3-N-GFPSpark was used to construct the HIF1ɑ-OE plasmid (HG11977-ANG), and pCMV3-C-FLAG for CD55-OE plasmid (HG10101-CF). The OE-plasmid or empty plasmid was transfected on 6/12/24-well plates in the presence of 10% fetal bovine serum using Lipo8000 (C0533, Beyotime, China).

**Neutrophil-induction of HL-60 cells and flow cytometry**

To induce neutrophil-like differentiation of HL-60 cells, 1.25% of DMSO was added to complete medium (20% FBS, RPMI1640) for consecutive 5 days. Complete medium and DMSO was replaced every 2 days. To further detect CD55 and CD11b expression in HL-60 cells after induction, 1×10^6^ cells were stained with 1 μL anti-CD11b-FITC, 1 μL anti-CD55-APC Abs in 100 μL FACS buffer at 4 ℃ for 30 min. After two washes these cells were ready for FACS analysis (NovoExpress, Agilent, United States).

**qRT-PCR analysis**

After 14 days of osteogenic induction, TRIzol was used to extract the total RNA of BMSCs, and the NanoDrop 8000 instrument (Thermo Fisher Scientific, United States) was used to detect the purity and concentration of RNA samples. Genomic DNA in total RNA sample was removed, and cDNA was synthesized by reverse transcription and stored at −20 ℃. Using cDNA as a template, reverse transcription quantitative polymerase chain reaction experiments were performed to detect the mRNA expression levels of Runx2, Sp7 and Bglap. For cells after siRNA transfection, RNA of BMSCs and HL-60 cells were extracted after 48 h of transfection. After reverse transcription, qRT-PCR was performed to detect mRNA expression levels of *Sting* and *Cd55*. Amplification reactions were set up in 20 μL reaction volumes containing SYBR Green PCR Master Mix (Vazyme, China), 1 μL volume of cDNA, and amplification primers. Primer sequences are listed in Supplementary Table 2.

**Western blotting**

mBMSCs, BM neutrophils and HL-60 cells were lysed in RIPA lysis buffer, PMSF and protein phosphatase inhibitors (Solarbio). After incubation at 4 ℃ for 30 min, protein lysates were processed by ultrasonic sonication and then centrifuged at 14,000×g for 15 min and the supernatant was collected. These protein lysates were boiled with 5×loading buffer and adjusted to a final protein concentration of 1 μg/μL. 20 μg of protein lysates were loaded in 10% Tris-glycine SDS polyacrylamide gel and transferred onto polyvinylidene difluoride (PVDF) membranes. Membranes were incubated with the primary Ab overnight at 4 ℃ and then with horseradish peroxidase-linked secondary antibody (Solarbio,1:2000) for 1 h at 4 ℃. The membranes were incubated with Enhanced Chemiluminescence (ECL) substrate, and targeted proteins were detected using an imaging system from Bio-Rad.
